# Exploration of promising optical and electronic properties of (non-polymer) small donor molecules for organic solar cells

**DOI:** 10.1038/s41598-021-01070-3

**Published:** 2021-11-02

**Authors:** Muhammad Khalid, Muhammad Usman Khan, Saeed Ahmed, Zahid Shafiq, Mohammed Mujahid Alam, Muhammad Imran, Ataualpa Albert Carmo Braga, Muhammad Safwan Akram

**Affiliations:** 1 Department of Chemistry, Khwaja Fareed University of Engineering & Information Technology, Rahim Yar Khan, 64200 Pakistan; 2 Department of Chemistry, University of Okara, Okara, 56300 Pakistan; 3 Institute of Chemical Sciences, Bahauddin Zakariya University, Multan, 60800 Pakistan; 4 Department of Chemistry, Faculty of Science, King Khalid University, P.O. Box 9004, Abha, 61413 Saudi Arabia; 5 Departamento de Química Fundamental, Instituto de Química, Universidade de São Paulo, Avenida Professor LineuPrestes, 748, São Paulo, 05508-000 Brazil; 6 School of Health and Life Sciences, Teesside University, Middlesbrough, TS1 3BA UK; 7 National Horizons Centre, Teesside University, Darlington, DL1 1HG UK

**Keywords:** Computational chemistry, Density functional theory

## Abstract

Non-fullerene based organic compounds are considered promising materials for the fabrication of modern photovoltaic materials. Non-fullerene-based organic solar cells comprise of good photochemical and thermal stability along with longer device lifetimes as compared to fullerene-based compounds. Five new non-fullerene donor molecules were designed keeping in view the excellent donor properties of 3-bis(4-(2-ethylhexyl)-thiophen-2-yl)-5,7-bis(2ethylhexyl) benzo[1,2-:4,5-c′]-dithiophene-4,8-dione thiophene-alkoxy benzene-thiophene indenedione (BDD-IN) by end-capped modifications. Photovoltaic and electronic characteristics of studied molecules were determined by employing density functional theory (DFT) and time dependent density functional theory (TD-DFT). Subsequently, obtained results were compared with the reference molecule BDD-IN. The designed molecules presented lower energy difference (ΔΕ) in the range of 2.17–2.39 eV in comparison to BDD-IN (= 2.72 eV). Moreover, insight from the frontier molecular orbital (FMO) analysis disclosed that central acceptors are responsible for the charge transformation. The designed molecules were found with higher λ_max_ values and lower transition energies than BDD-IN molecule due to stronger end-capped acceptors. Open circuit voltage (Voc) was observed in the higher range (1.54–1.78 V) in accordance with HOMO_donor_–LUMO_PC61BM_ by designed compounds when compared with BDD-IN (1.28 V). Similarly, lower reorganization energy values were exhibited by the designed compounds in the range of λ_e_(0.00285–0.00370 E_h_) and λ_h_(0.00847–0.00802 E_h_) than BDD-IN [λ_e_(0.00700 E_h_) and λ_h_(0.00889 E_h_)]. These measurements show that the designed compounds are promising candidates for incorporation into solar cell devices, which would benefit from better hole and electron mobility.

## Introduction

Environmental protection is in limelight as world has seen the record number of wild fires. United Nations has declared 2021–2030 as a ‘Decade on Ecosystem Restoration’ and the development of alternative sources of energy is at the forefront of this effort^1–3^. Solar power^4^ based on organic solar cells (OSCs) is considered efficient renewable energy source^5^ to cope with the global energy and environmental crisis and have the potential to overcome the limitations of silicon based solar cells, which are being heavy, rigid and unalterable HOMO–LUMO levels. The promising features of OSCs are solution-processability, flexibility and easy fabrication^5^. Over 14% power conversion efficiency (PCE) has been achieved by device optimization and due to innovation in photoactive materials such as non-fullerene solar cells based on single-junction polymer^6–8^. Many unique advantages including simple synthesis, high reproducibility, well-defined molecular structure and so on, are offered by small molecules as compared to polymers^9–13^. Small molecules having A-π-D-π-A type architecture incorporating two end-capped electron withdrawing units, two π bridges that interconnect the end terminal acceptor units with central core electron-donating unit offer exceptional photovoltaic performance (whether utilized as acceptors or donors) as compared to other donor–acceptor (D-A) types of small molecules^14–16^. For instance, Chen’s group have reported DR3TSBDT comprising of end-capped 3-ethylrhodanine unit and BDT substituted dialkylthiol as central core unit with 9.95% PCE^17^. A similar suit of small molecules is reported by Wei’s group claiming 11.08% PCE of small molecules comprising of end-capped fluorinated 1*H*-indene-1,3(2*H*)-dione units and central core of thiophene-substituted benzodithiophene unit^18^. Yan et al. in 2017 confirmed the well matched working of highly efficient donor material DR3TBDTT (DR3) with non-fullerene acceptors^19^. Hou’s groups suggested the new wide band gap donor that incorporates a two-dimensional trialkylthienyl-substituted benzodithiophene core building for efficient non-fullerene small molecule OSCs^20^. It has been demonstrated that LUMO level of acceptor materials and HOMO level of donor materials govern the open-circuit voltage (*V*oc) values^9,10^. Former research specifies that these LUMO and HOMO energy levels in turn depend on the electron accepting and donating capabilities of the acceptor and donor counterparts^21^. Thus, it can be predicted that by enhancing electron-accepting ability of entire molecule, superior *V*oc could be attained when the small molecule is used as the donor materials. Small molecule donors were developed by Adhikari et al. to replace the usual polymer donors used in solvent processed solar cells, like the molecular glasses^22^ or the T-Shaped Indan-1,3-dione derivatives^23^.

The BDD is a planar group enriched with electron-withdrawing capability and therefore extensively utilized for making alternate copolymer donor materials of D-A type^24^. Notably, Hou’s group developed PBDB-T polymer based on BDD unit is widely used in fullerene free polymer solar cells and has shown outstanding performance^25^. Zhang et al. described the synthesis of efficient small molecule donor BDD-IN of Acceptor2-π-Acceptor1-π-Acceptor2 (A_2_-π-A_1_-π-A_2_) sort incorporating of central unit BDD, thiophene-alkoxy benzene-thiophene to acts as the π-spacer, and indenedione as a terminal end-capped unit (A_2_) that showed 8.70% PCE with open circuit voltage to be 0.965 V^26^.

**Figure 1:**
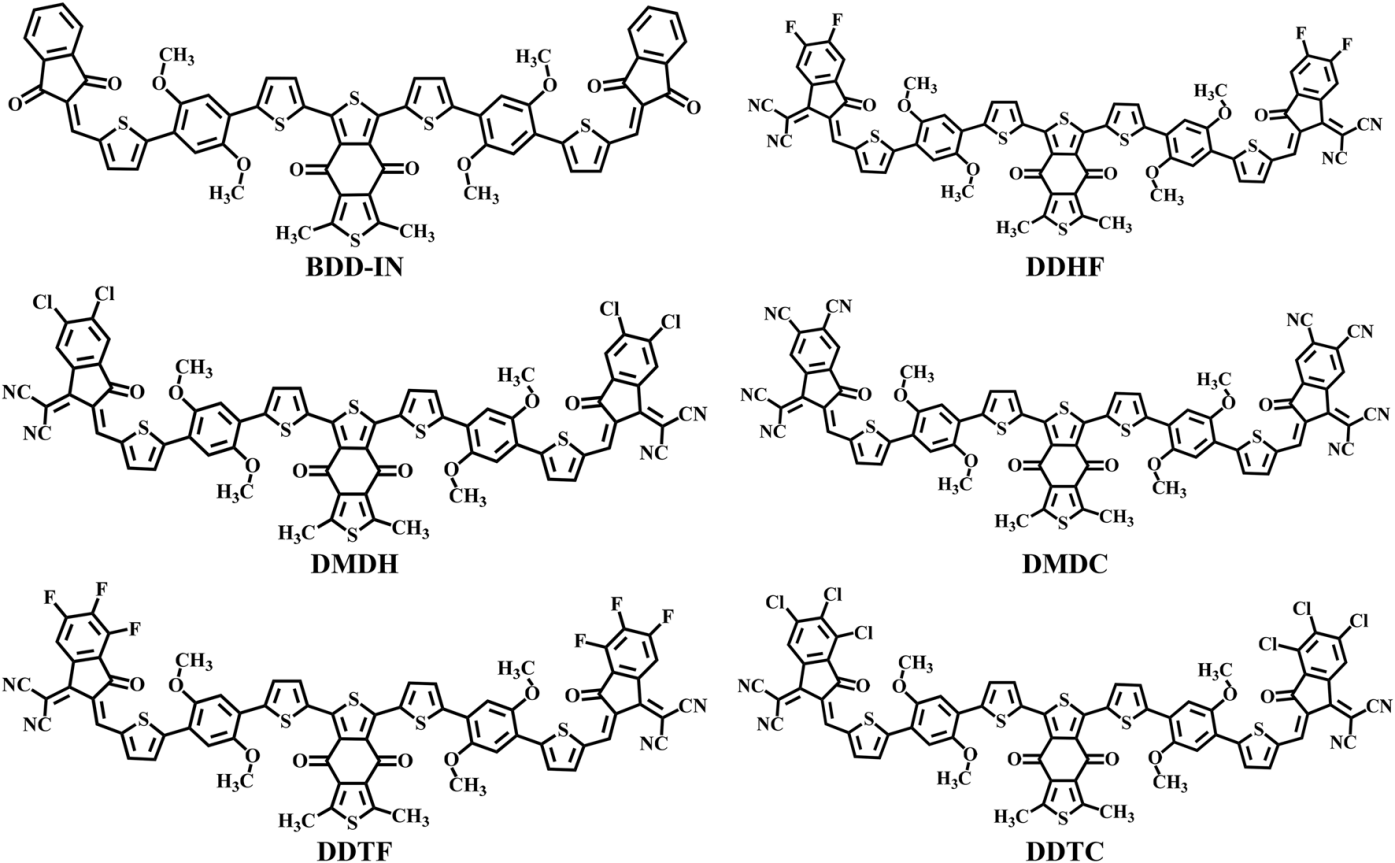
Structures of reference and designed compounds.

End-capped modification is seen as an efficient strategy to modify the optoelectronic properties of materials used in photovoltaic materials^27–31^. In this manuscript, this end-capped modification strategy is utilized on A_2_-π-A_1_-π-A_2_ type BDD-IN (dithieno[3,2-b:2′,3′-d]pyran) molecule^26^ and five new molecules comprising of different terminal end-capped acceptor units have been designed (Fig. 1). Comprehensive in-silico studies of designed molecules (DDHF, DMDH, DMDC, DDTF and DDTC) are presented with characterization of important parameters like binding energy, transition matrix density (TDM), frontier molecular orbital (FMOs) analysis, reorganizational energy of holes and electrons, excitation energy, charge transfer analysis, open circuit voltage (V_oc_) and compared with BDD-IN as a reference molecule. These new non-fullerene donors (NFDs) are predicted to have improved optoelectronic properties and potential to be used in the next generation of solar cells.

## Results and discussion

Reference molecule of A2-π-A1-π-A2-type recently synthesized by Zhang et al.^26^ is selected as BDD-IN in present quantum chemical investigation. The BDD-IN is composed of two indenedione (IN) terminal acceptor units (A2). These terminal acceptor units A2 are attached to central core unit BDD (A1) through a π-bridge (thiophene-alkoxy benzene-thiophene) units. Since, end-capped modification strategy is successfully utilized in literature to achieve the excellent photovoltaic parameters of optoelectronic materials^32–36^. Terminal acceptor unit (A2) indenedione (IN) of BDD-IN is modified by different highly efficient end-capped acceptors like 2-(5,6-difluoro-2-methylene-3-oxo-2,3-dihydro-1H-inden-1-ylidene)malononitrile, 2-(5,6-dichloro-2-methylene-3-oxo-2,3-dihydro-1H-inden-1-ylidene)malononitrile, 1-(dicyanomethylene)-2-methylene-3-oxo-2,3-dihydro-1H-indene-5,6-dicarbonitrile, 2-(4,5,6-trifluoro-2-methylene-3-oxo-2,3-dihydro-1H-inden-1-ylidene)malononitrile,2-(4,5,6-trichloro-2-methylene-3-oxo-2,3-dihydro-1H-inden-1-ylidene)malononitrile. The IUPAC names and codes of designed molecules are 2,2 ((2Z,2′Z)-((5,5′-((5,5′-(5,7-dimethyl-4,8-dioxo-4,8-dihydrobenzo[1,2-c:4,5-c′]dithiophene-1,3-diyl)bis(thiophene-5,2-diyl))bis(2,5-dimethoxy-4, 1-phenylene)) bis(thiophene-5,2-diyl))bis(methanylylidene))bis(5,6-difluoro-3-oxo-2,3-dihydro-1H-indene-2,1-diylidene))dimalononitrile (DDHF), 2,2′-((2Z,2′Z)-((5,5′-((5,5′-(5,7-dimethyl-4,8-dioxo-4,8-dihydrobenzo[1,2-c:4,5-c′]dithiophene-1,3-diyl)bis(thiophene-5,2-diyl))bis(2,5-dimeth oxy-4,1-phenylene))bis(thiophene-5,2-diyl))bis(methanylylidene))bis(5,6-dichloro-3-oxo-2,3-dih ydro-1H-indene-2,1diylidene))di-malononitrile (DMDH), (2Z,2′Z)-2,2′-((5,5′-((5,5′-(5,7-dimethy l-4,8-dioxo-4,8-dihydrobenzo[1,2-c:4,5-c′]dithiophene-1,3-diyl)bis(thiophene-5,2-diyl))bis(2,5-dimethoxy-4,1-phenylene))bis(thiophene-5,2-diyl))bis(methanylylidene))bis(1-(dicyanomethyle ne)-3-oxo-2,3-dihydro-1H-indene-5,6-dicarbonitrile) (DMDC), 2,2′-((2Z,2′Z)-((5,5′-((5, 5′-(5,7-dimethyl-4,8-dioxo-4,8-dihydrobenzo[1,2-c:4,5-c′]dithiophene-1,3-diyl)bis(thiophene-5,2-diyl))b is(2,5-dimethoxy-4,1-phenylene))bis(thiophene-5,2-diyl))bis(methanylylidene))bis(4,5,6-trifluor o-3-oxo-2,3-dih ydro-1H-indene-2,1-diylidene))di-malononitrile (DDTF), 2,2′-((2Z,2′ Z)-((5,5′-((5,5′-(5,7-dimethyl-4,8-dioxo-4,8-dihydrobenzo[1,2-c:4,5-c′]dithiophene-1,3-diyl)bis(thiophene-5,2-diyl))bis(2,5-dimethoxy-4,1-phenylene))bis(thiophene-5,2-diyl))bis(methanylylidene))bis(4,5, 6-tri-chloro-3-oxo-2,3-dihydro-1H-indene-2,1-diylidene))di-malononitrile (DDTC). The five new compounds mentioned here as DDHF-DDTC are designed as shown in Fig. 1.

**Figure 2:**
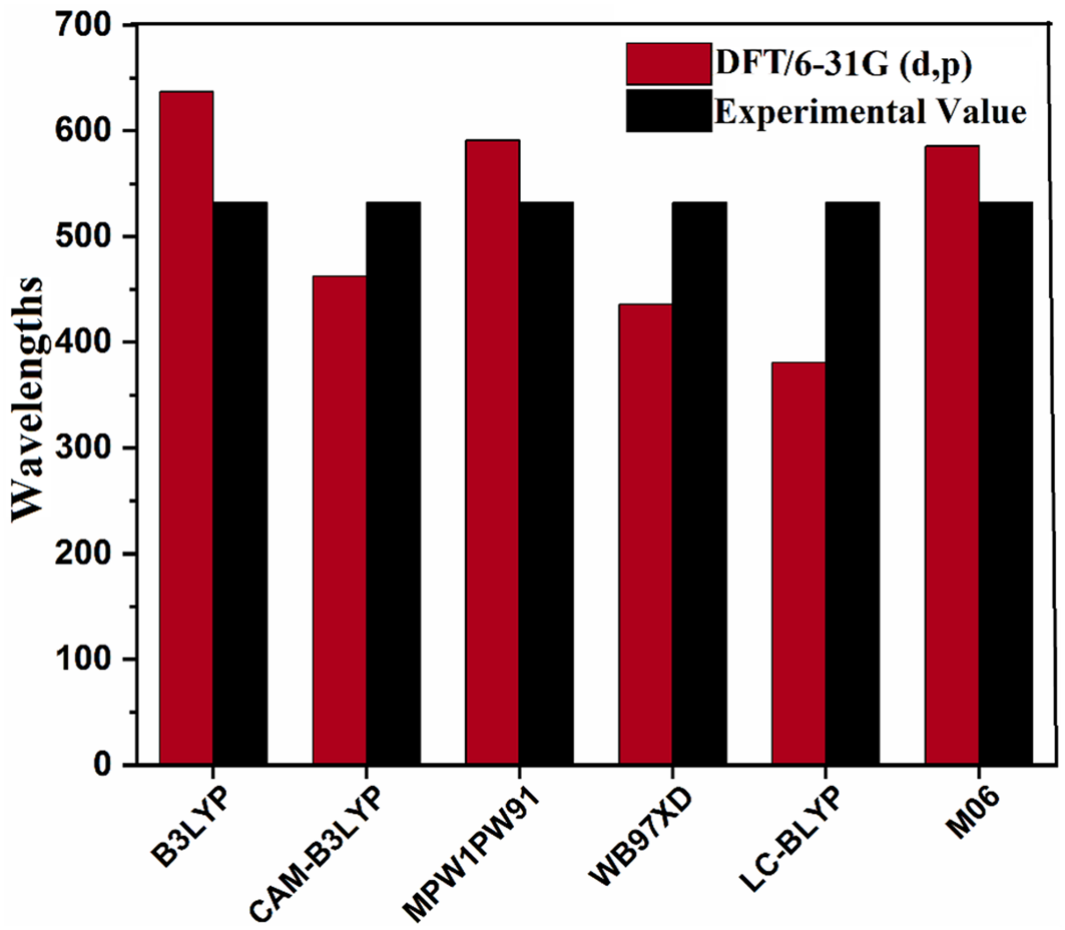
Graphical representation of comparison between experimentally and DFT calculated results of BDD-IN in chloroform (CHCl_3_) solvent at various functionals by utilizing origin 8.5 version (https://www.originlab.com/). All out put files of entitled compounds were accomplished by Gaussian 09 version D.01 (https://gaussian.com/g09citation/).

For selection of best suited functional of theory, λ_max_ values of BDD-IN molecule was calculated at six different functional including B3LYP/6-31G(d,p), CAM-B3LYP/6-31G(d,p), ωB97XD/6-31G (d, p), MPW1PW91/6-31G (d, p), M06/6-31G (d,p) and LC-BLYP/6-31G (d, p). The λ_max_ values of reference molecule on these levels are observed as 636.82 nm, 462.21 nm, 591.16 nm, 435.64 nm, 380.91 nm, and 585.49 nm respectively (Fig. 2). On comparing DFT computed values at different functionals with experimentally reported value of reference molecule (532 nm)^26^, it is apparent that results from M06/6-31G(d,p) level of theory correlate well with experimental value and can be used for further calculations of designed compounds (DDHF-DDTC) (see Fig. 2).

**Figure 3:**
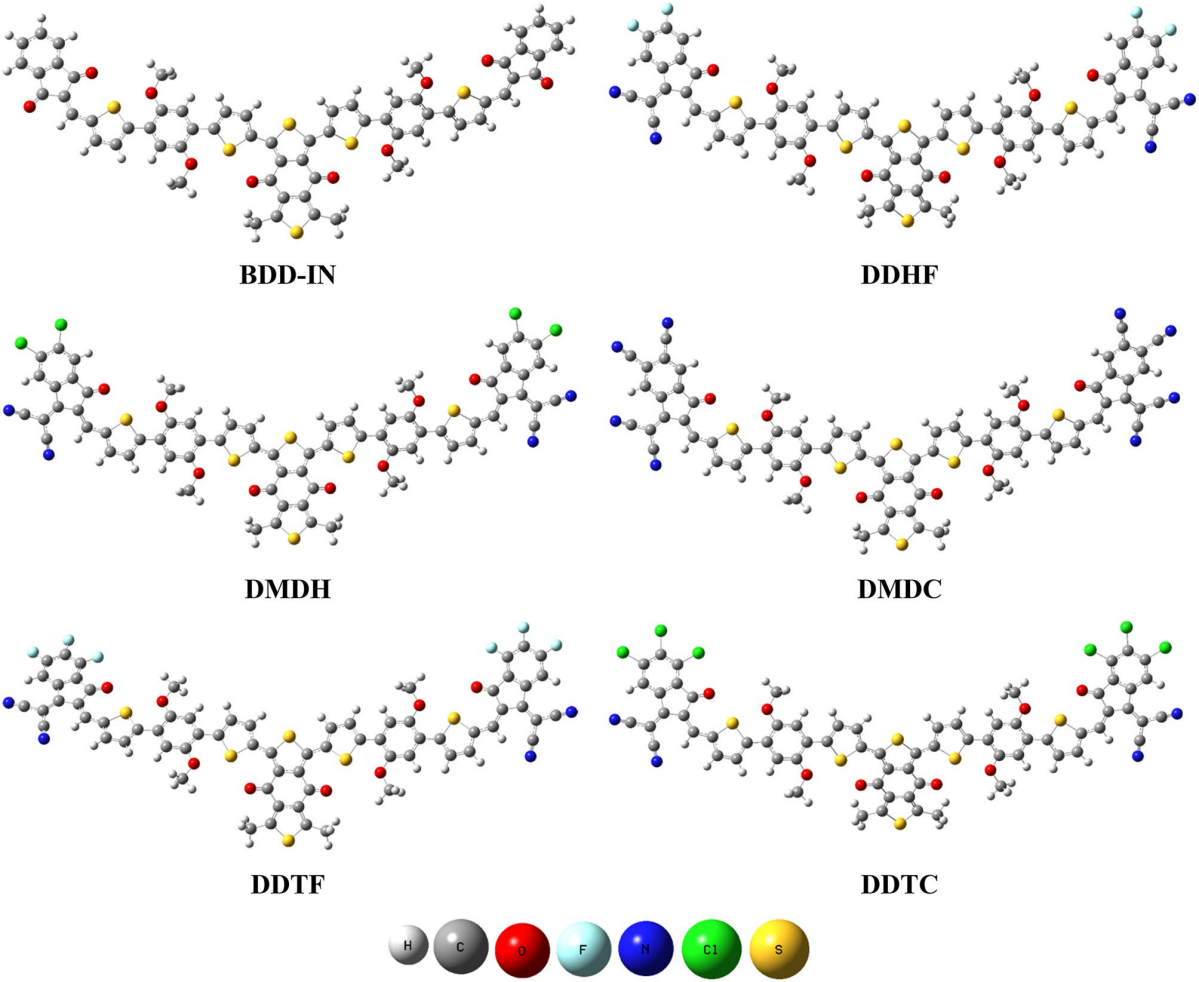
Optimized structures of studied compounds are made with the help of GaussView 5.0 and Gaussian 09 version D.01 (https://gaussian.com/g09citation/) Cartesian co-ordinates of all the molecules are presented in Supplementary Tables S1, S2, S3, S4, S5 and S6.

The optimized geometries found at true minima in potential energy surfaces are shown in Fig. 3.

### Frontier molecular orbitals (FMOs) analysis

**Figure 4:**
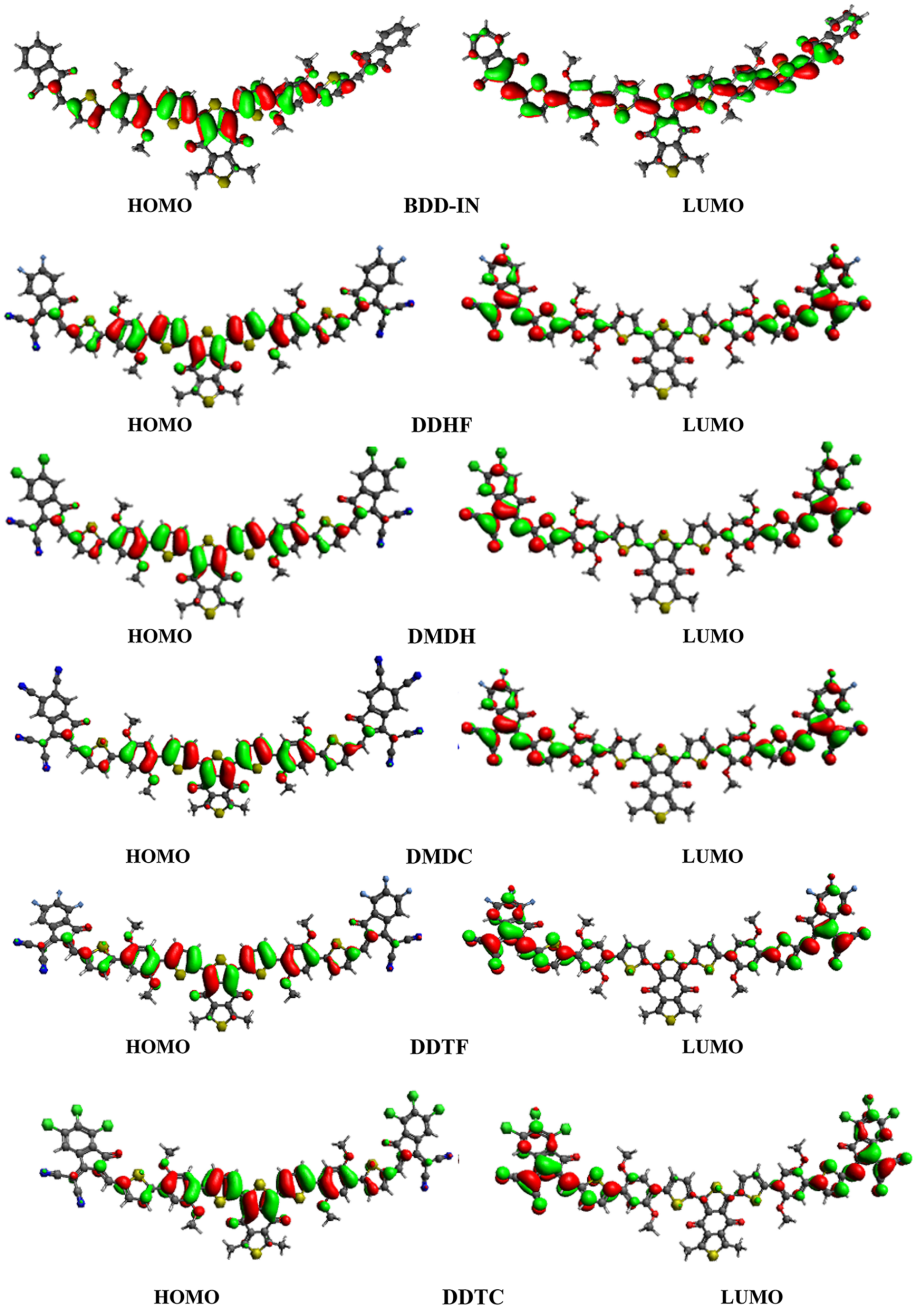
Pictographic representation of FMOs for reference and designed compounds. The pictures are drawn with the help of Avogadro software, Version 1.2.0. (http://avogadro.cc/). All out put files of entitled compounds were accomplished by Gaussian 09 version D.01 (https://gaussian.com/g09citation/).

FMOs analysis is a very useful tool for the characterization of electronic and optical properties of molecules^37^. According to band theory, HOMO and LUMO orbitals are denoted as valence and conduction band respectively. In photovoltaic materials, FMOs energy difference (ΔE = E_LUMO_-E_HOMO_) is considered as a hallmark of ability to carry charge^38–43^. The charge carrier mobility of designed donor molecules can be improved through conjugation due to the electronic delocalization within the molecular systems. Energy of HOMO, LUMO and their difference are fully coupled with PCE of solar cells. It is also illustrated that there is dynamic stability, electron transfer characteristics, chemical softness/hardness and reactivity of the designed compounds^44^. FMOs study for the distribution of charges and principally the ΔE between HOMO/LUMO orbitals is significant to recognize the electronic behavior and optical properties of the investigated compounds throughout the excitation process. FMOs study was performed at TD-DFT/M06/6-31G(d,p) level and HOMO, LUMO energies and their difference in energy ΔE) that are presented in Table 1. Additionally, the pictographic representation for FMOs for BDD-IN, and designed molecules are displayed in Fig. 4.

**Table 1:** FMO energies associated with designed and reference molecules.

Compound	HOMO	LUMO	ΔE = E_LUMO_ − E_HOMO_
BDD-IN	− 5.28	− 2.56	2.72
DDHF	− 5.54	− 3.15	2.39
DMDH	− 5.57	− 3.22	2.35
DMDC	− 5.78	− 3.61	2.17
DDTF	− 5.56	− 3.22	2.34
DDTC	− 5.58	− 3.27	2.31

*E* energy, ΔE *E*_LUMO_ − *E*_HOMO_.

The E_HOMO_ and E_LUMO_ of DMDC are less than all other molecules, signifying the better electron withdrawing effect of the four terminal cyano units of DMDC. Conversely, HOMO–LUMO values in BDD-IN were found greater as compared to the corresponding value of designed structures that predicts the lesser efficiency than the end-caped acceptors of all designed compounds. Moreover, in DMDC, the HOMO–LUMO energy levels are stable than DDHF and DMDH, which designate the significant proficiency of end-caped acceptor moieties. Furthermore, HOMO–LUMO values of DDTF and DDTC were observed to be larger than DDHF and DMDH values due to the end-caped acceptors of DDTF and DDTC, respectively. Overall, the HOMO–LUMO values are found in the following reducing order: BDD-IN > DDHF > DDTF > DMDH > DDTC > DMDC and BDD-IN > DDHF > DMDH > DDTF > DDTC > DMDC, respectively.

The ΔE is another substantial means to signify the charge transformation within a molecule. The maximum ΔE value (2.72 eV) amongst all investigated molecules is seen in BDD-IN. Table 1 shows that ΔE values are diminished for DMDC as compared to BDD-IN, DDHF, DMDH, DDTF and DDTC. Among designed and the reference compounds, DMDC exhibits the lowest energy gap value which could be attributed to the combined effect of extended conjugation and on better electron withdrawing effect of the four terminal cyano units present in end-capped acceptor of DMDC as compared to other compounds. The ΔE result of all the studied compounds are reported in the region of 2.17–2.72 eV. As indicated in Fig. 4, the HOMO charge is propagated on the central accepter moiety and a little amount of charge is observed on the π-spacer, whereas LUMO is dispersed on the end-capped acceptor units of the studied compounds. This charge dispersion patterns show that occurrence of electrons delocalization is caused by high donor to acceptor charge transfer with the aid of π-bridging unit.

**Figure 5:**
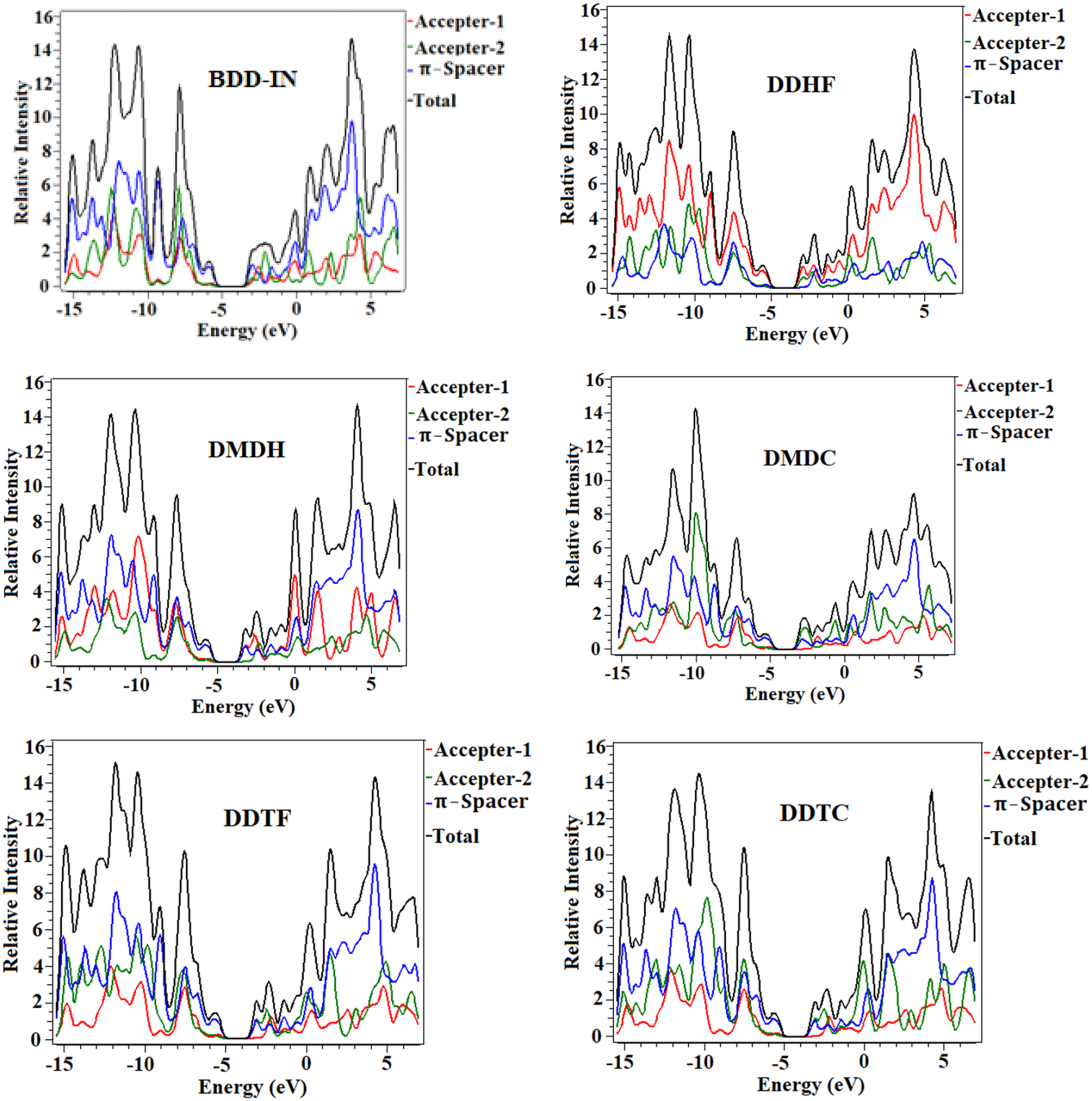
Graphical representation of the density of states (DOS) for BDD-IN, DDHF-DDTC drawn by utilizing PyMOlyze 1.1 version (https://sourceforge.net/projects/pymolyze/). All out put files of entitled compounds were accomplished by Gaussian 09 version D.01 (https://gaussian.com/g09citation/).

Partial density of states (P-DOS) were computed at M06/6-31G (d, p) level of DFT (Fig. 4). Figure 5 is also in accordance with the factors presented in FMOs study and along with Fig. 4, it reveals that charge is concentrated around LUMO and HOMO because of strong dragging and the electron accepting capability of terminal units. In BDD-IN, the HOMO charge density is occupied primarily on central acceptor part (A1) and π-spacer, while the LUMO is occupied completely on whole molecule except upper part of the central acceptor unit (A1) and half portion of the end-caped acceptor (A2). The HOMO charge density is completely distributed on the central acceptor part (A1) and π-spacer unit and the LUMO is distributed completely on the end-capped acceptor units A2. Overall, these charge density circulation patterns reveal that electron delocalization is happened and huge charge transferred from the central acceptor part (A1) to end-capped acceptor units A2 with the assistance of the bridge part occurred.

### Optical properties

**Figure 6:**
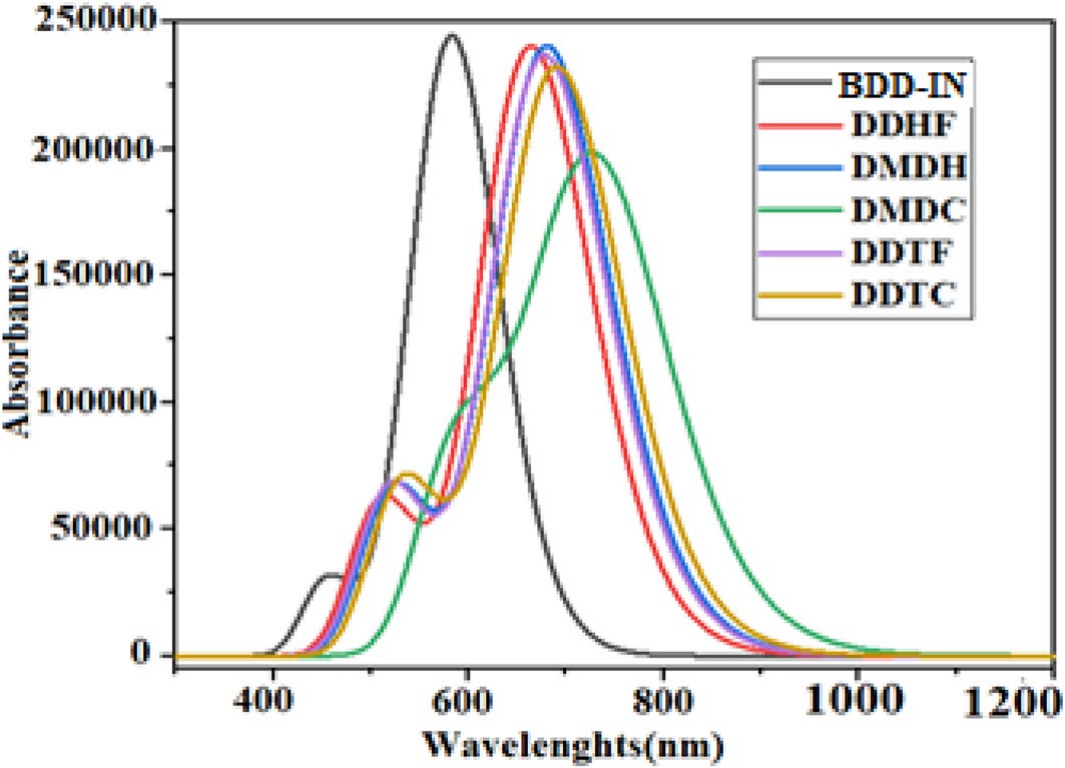
Graphical representation of UV–Visible absorption spectra of studied compounds.

To evaluate the photo-physical responses for BDD-IN, DDHF-DDTC, TD-DFT at M06/6-31G(dp) level of theory was employed to execute UV–Visible absorption spectra in CHCl_3_ solvent. The computed results attained from spectral analysis comprising $\lambda_{{{\text{max}}}}$, transition energy (E_x_), oscillator strengths ($f_{os}$), and transition natures of the investigated molecules are arranged in Table 2. Absorption spectra are presented in Fig. 6 showing two absorption peaks for each studied compound representing the major and minor absorption peaks.

**Table 2:** Wavelength, excitation energy and oscillator strength of studied compounds.

Compound	DFT $\lambda$(nm)	Exp. $\lambda$(nm)	E (eV)	*f*	MO contributions
BDD-IN	585	532	2.11	3.2	H-1 → L + 1(11%), H → L (80%)
DDHF	666		1.86	3.2	H-1 → L + 1(12%),H → L(80%)
DMDH	683		1.81	3.2	H-1 → L + 1(12%),H → L(80%)
DMDC	732		1.69	2.6	H-1 → L + 1(10%),H → L(82%)
DDTF	679		1.82	3.0	H-1 → L + 1(12%),H → L(80%)
DDTC	692		1.79	3.0	H-1 → L + 1(12%),H → L(81%)

*f* = oscillator strength, MO = molecular orbital, H = HOMO, L = LUMO.

Maximum absorption peak ($\lambda_{{{\text{max}}}}$** = **585 nm) of BDD-IN is in good agreement with experimental $(\lambda_{{{\text{max}}}}$ = 532 nm) value as can be seen in Table 2. The electron withdrawing groups can potentially be attributed for the red shift in $\lambda_{{{\text{max}}}}$ values in the observed spectra. The TD-DFT based calculations also reveal that absorbance of all studied compounds is found in the visible region within the range of 666–732 nm. The $\lambda_{{{\text{max}}}}$ values of designed compounds are reported considerably larger and red shifted than that of BDD-IN molecule (Fig. 6). Table 2 reveals that the lowest value of $\lambda_{{{\text{max}}}}$ is found in BDD-IN molecule among all the investigated compounds. The strong electron withdrawing capability of four F atoms and four CN groups present in end-capped unit of the compound DDHF successfully caused the red shift to $\lambda_{{{\text{max}}}}$ value to 666 nm which confirms the utility of DDHF end-capped acceptor as compared to other end-capped acceptors of BDD-IN molecule which lack F and CN groups. Replacement of four fluorine with chlorine atoms in the end-capped acceptor of DDHF converts it to the compound DMDH. This change increases the $\lambda_{{{\text{max}}}}$ value to 683 nm showing potential of DMDH over DDHF and BDD-IN molecules. Similarly, the replacement of four chloro groups with cyano groups in DMDH yields compound DMDC, where $\lambda_{{{\text{max}}}}$ value shifts to 732 nm, the largest $\lambda_{{{\text{max}}}}$ value among all the designed compounds. These results confirmed the superiority of DMDC end-capped acceptor containing CN units over all other compounds. The compound DDTF where end-capped unit contains six F atoms successfully shifted the $\lambda_{{{\text{max}}}}$ value to 679 nm, showing that the number of fluorine atoms play their role in causing the red shift. Likewise, the replacement of six fluoro atoms with chloro groups in DDTF produced compound DDTC which also led to the successful red shift of the maximum absorption peak ($\lambda_{{{\text{max}}}}$) value to 692 nm and proved the usefulness of DDTC over DDTF molecule as well as over BDD-IN molecule. Overall, the designed compounds showed red shift of 81, 98, 147, 94 and 107 nm in comparison to that of BDD-IN molecule correspondingly. The maximum absorption peak ($\lambda_{{{\text{max}}}}$) for BDD-IN molecule and designed compounds is in the following escalating order: BDD-IN < DDHF < DDTF < DMDH < DDTC < DMDC.

Excitation energy or charge transfer character exhibits valuable insights and proposes that molecules having smaller transition energy accommodate higher charge transfer capability, easy excitation between the HOMO to LUMO and possess higher PCEs. In case of BDD-IN, maximum value of excitation energy is noticed as 2.11 eV. Strong electron accepting capability of end-caped groups reduces the excitation energy in designed compounds. Hence, the calculated transition energy values show that the reference molecule BDD-IN has greater value of transition energy than the designed compounds. The excitation energy values for DDHF-DDTC are found to be 1.86, 1.81, 1.69, 1.82 and 1.79 eV*,* respectively. The lowest excitation energy is 1.69 eV in the case of DMDC due to presence of cyano group and extended conjugation. The increasing order for excitation energy of the designed compounds agrees with the decreasing $\lambda_{{{\text{max}}}}$ order: BDD-IN > DDHF > DDTF > DMDH > DDTC > DMDC. The smallest transition energy of DMDC and the highest $\lambda_{{{\text{max}}}}$ value make it a suitable candidate to be used in solar cells due to the better optoelectronic properties. The previous examination concludes that all the designed molecules containing higher $\lambda_{{{\text{max}}}} \varvec{ }$ and lower transition energy values possess good potential of optoelectronic properties than that of BDD-IN molecule. Hence, all designed compounds especially DMDC is predicted to be capable of being utilized as an electron donating molecule in OSCs applications.

### Reorganization energy (RE)

Reorganization energy is a fundamental characteristic to correlate the molecular structure with charge transportation ability of compounds which can help to design the best candidates for photovoltaic applications. The charge transfer capability and reorganization energy are inversely proportional to each other. To evaluate the transportability of charge by designed molecules: DDHF, DMDH, DMDC, DDTF and DDTC, the reorganization n energies were calculated by utilizing Eqs. (3) and (4). Reorganization energy of all studied compounds was computed at the M06/6-31G (d, p) level of theory (Table 3).

**Table 3:** Reorganization energy of BDD-IN, DDHF-DDTC molecules.

Molecule	$\lambda_{e}$ (*E*_*h*_)	$\lambda_{h}$ (*E*_*h*_)
BDD-IN	0.00700	0.00889
DDHF	0.00370	0.00826
DMDH	0.00333	0.00802
DMDC	0.00285	0.00847
DDTF	0.00343	0.00824
DDTC	0.00311	0.00820

$\lambda_{{\text{e}}}$: Rate of transfer of electrons, $\lambda_{{\text{h}}}$: rate of transfer of hole.

The anionic and cationic geometries indicate the transformation of electron and hole towards acceptor from the donor molecule. Reorganization energy (RE) can be utilized to compute the charger transfer (CT) between the electron donating and accepting moieties. This energy is categorized in two segments: internal reorganization energy (RE-$\lambda_{{{\text{int}}.}}$) and external RE (${\varvec{\lambda}}_{{{\text{ext}}.}}$). Both $\varvec{ \lambda }_{{{\text{ext}}.}}$ and ${\varvec{\lambda}}_{{{\text{int}}.}}$ specifies the polarization effect on the external environmental and the rapid alterations in the internal geometry, respectively. For this manuscript, the environmental variations have not been considered as they have little effect and only $\varvec{ \lambda }_{{{\text{int}}.}}$ is considered. The value of $\varvec{ \lambda }_{{\text{e}}}$ for DDHF (0.0037 *E*_*h*_) was found to be less than BDD-IN (0.0070) signifying the dominant electron transfer rate for DDHF as compared to BDD-IN. Likewise, the value of $\varvec{ \lambda }_{{\text{e}}}$ for DMDH (0.00333 *E*_*h*_) was noticeably smaller than BDD-IN (0.0070 *E*_*h*_) and DDHF implying that two terminal chlorine groups work efficiently to tune the intra molecular charge transfer as compared to two fluorines in DDHF. Due to cyano groups modification in DMDC, least value of electron reorganization energy was found to be 0.00285 *E*_*h*_ among all the studied compounds indicating the best efficiency of cyano groups as compared to other functional groups present in terminal acceptors. The $\varvec{ \lambda }_{{\text{e}}}$ for DDTF and DDTC were also found smaller than the reference molecule due to end-capped modifications.

The highest ${\varvec{\lambda}}_{{\text{h}}}$ value of all investigated compounds was noted and compared with BDD-IN molecule. As discussed, the reasons for reduction in electron reorganization energy, hole reorganization energy is also abridged in designed compounds due to end-capped modifications of different functional units. The ${\varvec{\lambda}}_{{\text{h}}}$ values of designed compounds DDHF-DDTC are 0.00826, 0.00802, 0.00847, 0.00824 and 0.00820 *E*_*h*_ respectively that are much smaller as compared to that of BDD-IN (0.00889 *E*_*h*_) and this reveals that designed compounds have a greater rate of transformation of holes in comparison to BDD-IN molecule. Overall, the values of $\varvec{ \lambda }_{{\text{e}}}$ are smaller as compared to $\varvec{ \lambda }_{{\text{h}}}$ which proposes that these compounds are inspiring candidates for transfer of electrons.

### Open circuit voltage (Voc)

Voc is significant to illustrate the execution of OSCs^45^ and reveals the highest value of current that can be taken away from an optical device in this context^46^. It is at the point of maximum voltage that can be gained at zero current value from any device. Voc depends on numerous features: external fluorescence proficiency, charge carrier recombination, light source, temperature of OSCs device, light intensity, and different environmental features. Principally, Voc relies on the saturation current and light generation that assists the recombination in the devices. Voc is approximately proportional to $\Delta {\text{E}}$ of donor and acceptor molecules corresponding to HOMO_donor_–LUMO_PC61BM_ energies. In the current quantum chemical evaluation, Voc values are calculated by utilizing acceptor molecule (PC_61_BM). The Voc of PC_61_BM is variable and depends completely on the electron donating compound. Theoretically the calculated results of Voc for the OSCs have been with the aid of Eq. (1), established by Scharber and his co-workers^47^.

1 $$ {\text{Voc}} = \left( {\left| {{\text{E}}_{{{\text{HOMO}}}}^{{\text{D}}} } \right| - \left| {{\text{E}}_{{{\text{LUMO}}}}^{{\text{A}}} } \right|} \right) - 0.3 $$

**Figure 7:**
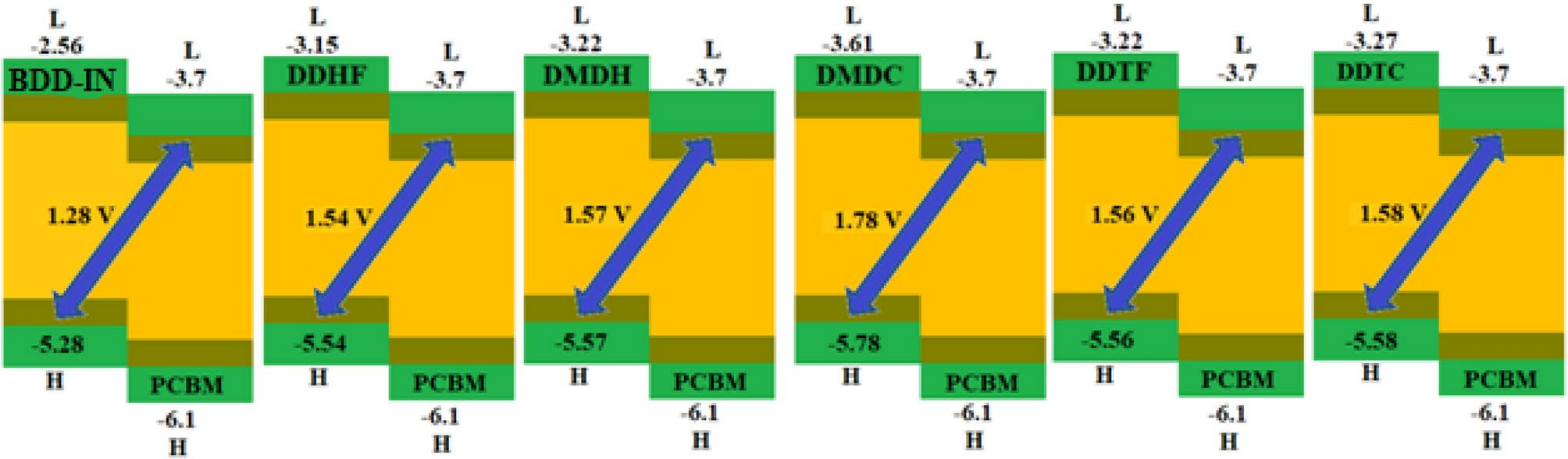
Graphical representation of Voc for studied compounds. All out put files of the designed compounds were accomplished by Gaussian 09 version D.01 (https://gaussian.com/g09citation/).

The HOMO energy levels of the studied molecules in comparison with LUMO energy level of well-known acceptor material PC_61_BM are shown in Fig. 7.

The Voc value for compound BDD-IN with regards to HOMO_donor_–LUMO_PC61BM_ energy difference is calculated to be 1.28 V, while designed compounds with ${\text{LUMO}}_{{{\text{PC}}61{\text{BM}}}}$ energy difference shows higher Voc values of 1.54, 1.57, 1.78, 1.56 and 1.58 V, correspondingly in comparison to the BDD-IN molecule. The smaller HOMO level of designed molecules as compared to BDD-IN is the major cause for higher value of Voc. The HOMO energy levels of DDHF-DDTC are noticed to be in following reducing order: BDD-IN > DDHF > DDTF > DMDH > DDTC > DMDC. The Voc value of DDHF was calculated to be 0.26 V which is greater than the value for BDD-IN molecule. Likewise, in DMDH, Voc was calculated to be 0.3 V larger as compared to the BDD-IN molecule. The largest Voc value (1.78 V) among all the investigated molecules is computed in case of DMDC that was found to be 0.50 V higher than that of BDD-IN molecule. Likewise, Voc for DDTF is calculated to be 0.28 V larger as compared to BDD-IN molecule and larger than DDHF but less than DMDH and DMDC molecules. Moreover, the Voc value of DDTC is calculated as 0.3 V higher as compared to BDD-IN molecule and greater than DDHF and DMDH molecules but less than DMDC molecule. The least value of Voc 1.54 V amongst all designed compounds was found in the case of DDHF which was still 0.26 V higher than BDD-IN (1.28 V) value. This investigation proves that all designed molecules DDHF-DDTC have the potential to be suitable materials for OSCs applications when blended with well-known acceptor polymer PC_61_BM.

### Charge transfer analysis

**Figure 8:**
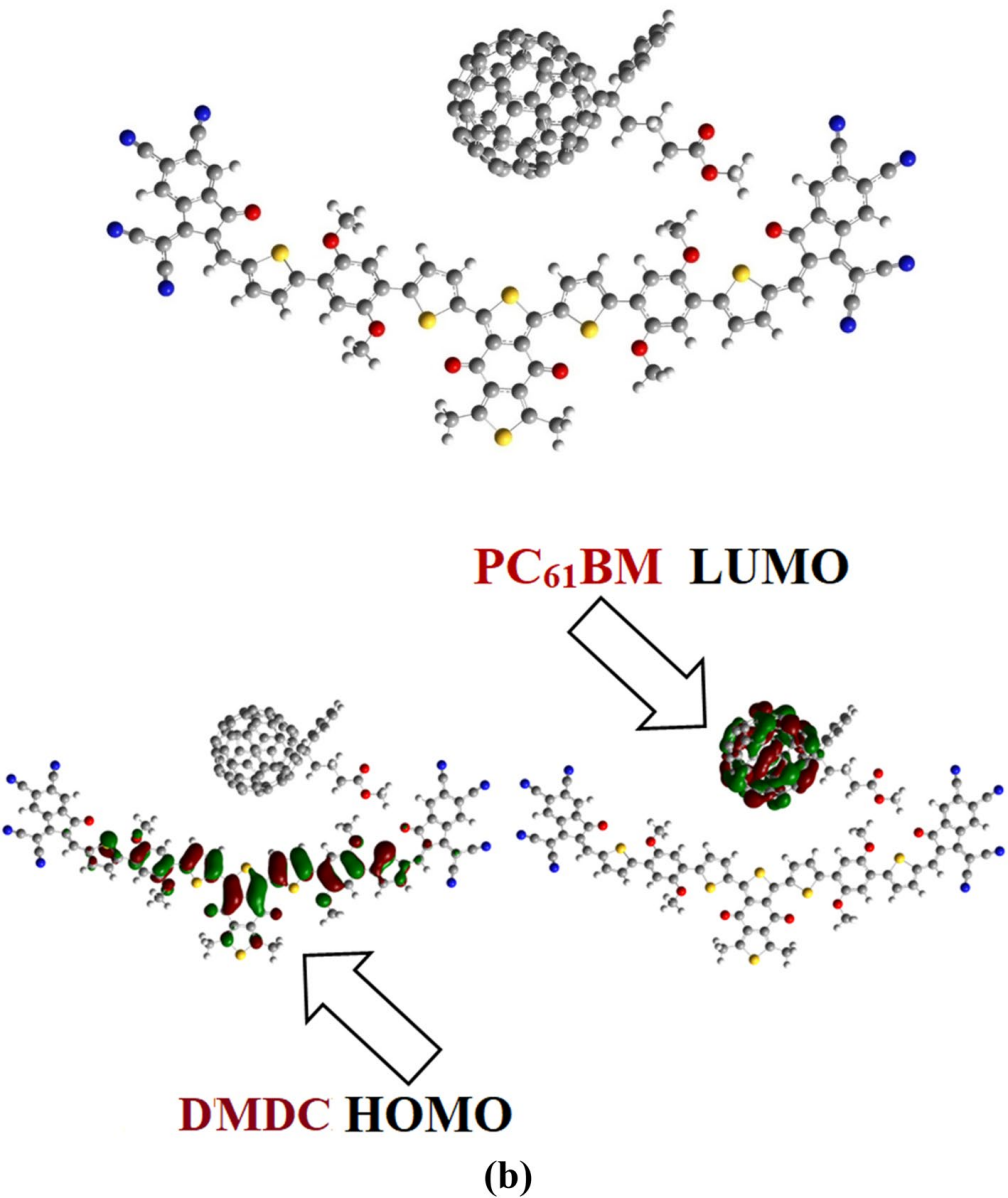
Graphical representation of optimized geometry of DMDC: PC_61_BM and Charge transfer between ${\text{HOMO}}_{{{\text{donor}}}}$ to ${\text{LUMO}}_{{{\text{PC}}61{\text{BM}}}}$.These exhibits are prepared with the help of GaussView 5.0 and Gaussian 09 version D.01 (https://gaussian.com/g09citation/).

In the charge transfer (CT) investigations, a complex is established between DMDC and well-known acceptor polymeric materials, in this study we are using PC_61_BM. Optimized geometry of the DMDC: PC_61_BM complex is shown in Fig. 8. The interactions between donor molecule DMDC and acceptor polymeric material PC_61_BM interact at various points, C3 and polymer sides are parallel. Whereas functional group side of PC_61_BM is positioned to the end-capped acceptor of DMDC, whereas ball side of PC_61_BM points toward the core side of the DMDC molecule (Fig. 8).

The electronic cloud of DMDC: PC_61_BM arrangement is majorly influenced by the relative positioning of the DMDC and PC_61_BM which eases the charge transformation between the electron donating and accepting parts. Dipole moment of the complex largely comes from DMDC to the acceptor and acts as the cause for effective exciton dissociation at the DMDC: PC_61_BM boundary^48–50^. The dipole moment is complex because of the electrostatic interactions of permanent dipole moment of PC_61_BM with respect to DMDC. Existing literature supports that the polymer part is largely responsible for the dipole moment within the complex, where, the dipole moment vector originates from the polymer side and point towards the core of the DMDC molecule. The HOMO–LUMO electronic structure and charge circulation pattern were computed at the M06/6-31G(d,p) level of DFT. The HOMO charge concentration in DMDC: PC_61_BM complex is concentrated on the central part and in part on the π-spacer of the donor DMDC molecule (Fig. 8b), while the LUMO charge is dispersed on end-capped group polymer PC_61_BM (Fig. 8b). The orbital diagram illustrates that the HOMO-to-LUMO excitation is a charge transferred from the electron donating DMDC to the electron accepting PC_61_BM molecule. The transformation of charge concentration from the electron donating molecule to the electron accepting is an indication of a good photovoltaic material.

### Exciton binding energy (*E*_*b*_) and transition density matrix (TDM)

**Figure 9:**
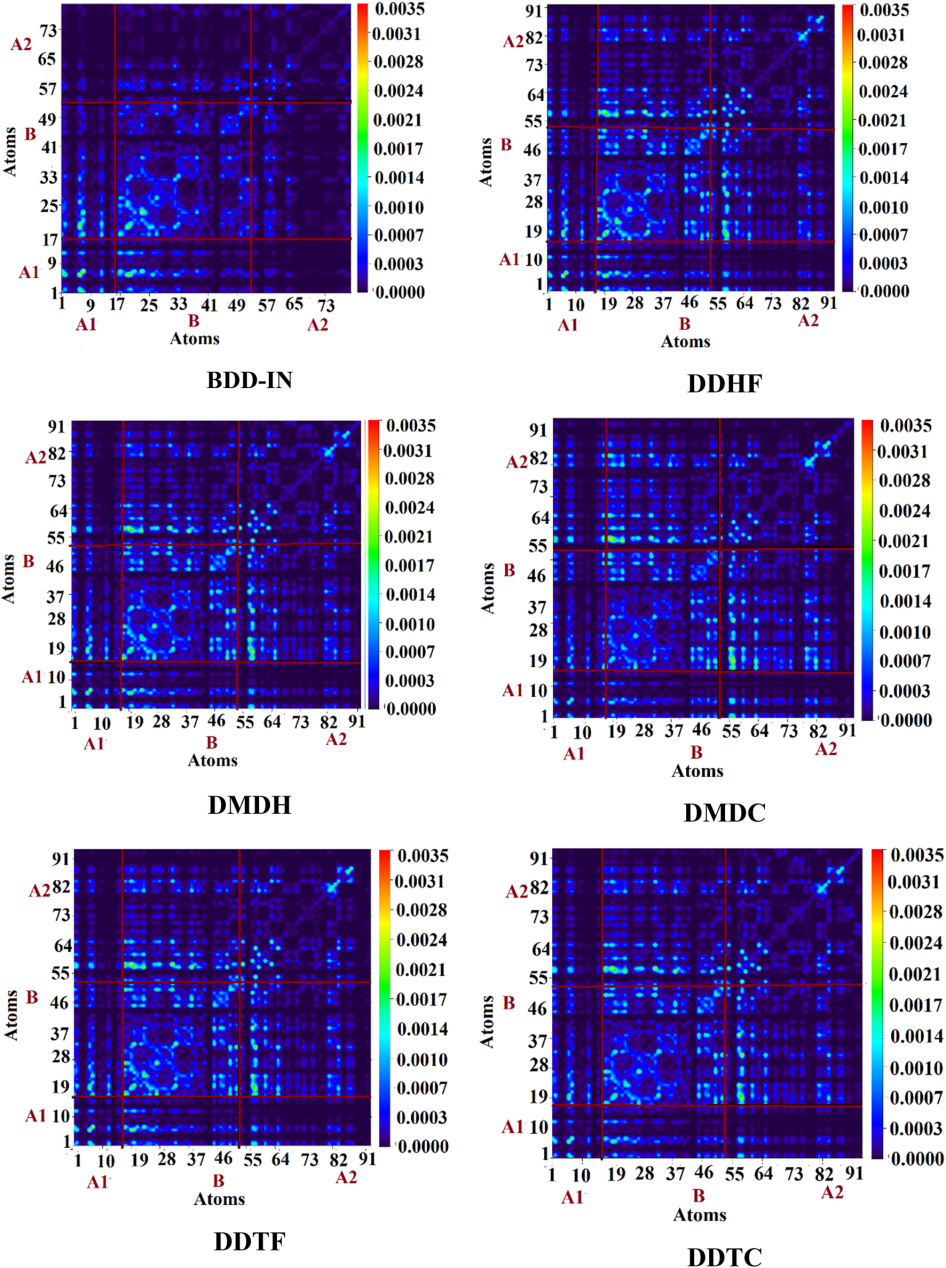
Graphical representation of transition density matrix (TDM) at the S1 state. The were drawn with the help of Multiwfn 3.7 software (http://sobereva.com/multiwfn/). All out put files of designed compounds were accomplished by Gaussian 09 version D.01 (https://gaussian.com/g09citation/).

The transitions nature is assessed by calculating the transition density matrixes (TDMs). The M06/6-31G (d, p) level of theory was utilized to calculate the emission and absorption of the S1 state in vacuum, the results are exhibited in Fig. 9. Due to the minute contribution in transitions, the influence of hydrogen atoms is overlooked. TDMs technique allows us to calculate, (1) the interaction within electron donating and accepting moieties in the excited state; (2) the electronic excitation (3) electron hole localization. For the determination of these properties, we distributed our studied molecules into three parts namely, acceptor-1 (A1), π-bridge (B) and acceptor-2 (A2).

The TDMs graphs designate that all compounds show analogous behavior in which electron coherence is majorly accessible on the diagonal of π-bridge segment B and a minute portion is present on A1. Similarly, in case of all the designed molecules a major portion of charge is present on the end-capped acceptor A2, while in case of reference molecule BDD-IN, a minute portion is seen on A2. The electron coherence in designed molecules display similar trend that is, majorly present on the π-bridge segment B and end-capped acceptor A2, while a minute portion is present on A1. The TDMs graph for BDD-IN shows that major portion of electron coherence is present on the π-bridge segment B and a minute portion is present on A1, while a minute portion of electron coherence is seen on a diagonal of A2. From TDM diagrams, as shown in Fig. 9, the electron coherence of BDD-IN, DDHF-DDTC confirm that the electrons are successfully transferred from the central acceptor A1 to the π-bridge and lastly the electron charge concentration moves to the electron acceptors. Moreover, the coefficient of interaction between donor and acceptor groups are in order of BDD-IN > DMDH > DDHF > DDTF > DDTC > DMDC. This order suggests that the connection of the hole and the electrons of DMDC may be weaker as compared to the remaining studied compounds, however this exhibited greater and easier exciton dissociation in the excited state.

### Binding energy (Eb)

The binding energy profile shows that DMDC exhibit maximum charge dissociation potential of all the investigated compounds and overall the charge dissociation capability for all the designed compounds is found to be higher than that of BDD-IN molecule suggesting that designed molecules would indeed enhance the current charge concentration (*Jsc*). Binding energy (*E*_*b*_) is an important factor that supports the assessment of the optoelectronic properties of OCs. Binding energy enables us to calculate the interaction of the columbic forces among the hole and electron. The *E*_*b*_ and columbic interaction of the hole and electron are directly proportional with each other and both have inverse relation with exciton dissociation in the excited state. *E*_*b*_ of BDD-IN, DDHF-DDTC molecules are calculated by utilizing the following Eq. (4).

2 $$ E_{b} = E_{{{\text{H}} - {\text{L}}}} - E_{{{\text{opt}}}} $$

In Eq. (2), ${\text{E}}_{{{\text{HOMO}} - {\text{LUMO}}}}$ signifies energy difference of HOMO/LUMO and ${\text{E}}_{{{\text{opt}}}}$ shows that the smallest quantity of energy required for the first excitation (gained from S_0_ to S_1_), by producing pair of the electron and hole^51,52^. Calculated results for binding energy (*E*_*b*_) are arranged in Table 4.

**Table 4:** Calculated ${\text{ E}}_{{{\text{H}} - {\text{L}}}}$, ${\text{E}}_{{{\text{opt}}}}$, and ${\text{E}}_{{\text{b}}}$ of BDD-IN, DDHF-DDTC.

Molecule	${\text{E}}_{{{\text{HOMO}} - {\text{LUMO }}}}$ (eV)	${\text{E}}_{{{\text{opti}}}}$ (eV)	${\text{E}}_{{{\text{binding}}}}$ (eV)
BDD-IN	2.34	2.11	0.23
DDHF	2.39	1.86	0.53
DMDH	2.35	1.81	0.54
DMDC	2.17	1.69	0.48
DDTF	2.34	1.82	0.52
DDTC	2.31	1.79	0.52

The ${\text{E}}_{{\text{b}}}$ value of BDD-IN molecule is 0.23 eV* and it is exciting to note* that the binding energy (E_b_) values of DDHF-DDTC molecules, higher in comparison to the BDD-IN molecule, are found to be 0.53, 0.54, 0.48, 0.52 and 0.52 eV correspondingly. Descending order of *E*_*b*_ values for investigated compounds is DDTC > DMDH > DDHF > DDTF > DMDC > BDD-IN. These results with maximum *E*_*b*_ value of DDHF-DDTC molecules and minimum *E*_*b*_ value of BDD-IN molecule are in good agreement with TDMs.

## Conclusions

A series of new non-fullerene donors (DDHF-DDTC) are designed keeping in view a synthesized non-fullerene-based electron donor molecule (BDD-IN) with Acceptor-π-Acceptor-π-Acceptor (A-π-A-π-A) configuration using different end-capped electron accepting groups. The optoelectronic properties of designed compounds are found to be better than that of BDD-IN molecule. The designed and reference compounds exhibited lower energy difference in the region of 2.17–2.39 eV and 2.72 eV, respectively and revealed that results of designed compounds are better than that of the reference molecule. Interestingly, DMDC exhibited the smaller energy difference of 2.17 eV value which is obtained less in magnitude than designed molecules as well as BDD-IN molecule due to the strong electron withdrawing effect of the end-capped acceptor. Moreover, DDHF-DDTC molecules exhibited red shifted $\lambda_{max}$ in visible range with 666–732 nm values in comparison to BDD-IN (585 nm). Similarly, to energy difference pattern, DMDC is reported with smaller transition energy value (1.69 eV) along with absorption peak value as 732 nm. Open circuit voltage (Voc) with respect to the HOMO_Donor_– LUMO_PC61BM_ of DDHF-DDTC, are found in the region of 1.54–1.78 V markedly greater when compared with BDD-IN (Voc = 1.28 V). DMDC molecule is the promising candidate as consisting of the lower values of ${\varvec{\lambda}}_{{\text{e}}}$ and ${\varvec{\lambda}}_{{\text{h}}}$ transportation:$\varvec{ \lambda }_{{\text{e}}}$ (0.00285 *E*_*h*_) and ${\varvec{\lambda}}_{{\text{h}}}$ (0.00847 *E*_*h*_) among all the investigated compounds. Finally, among all DDHF-DDTC, the designed molecule DMDC presented exceptional optoelectronic properties because of the strong electron withdrawing effect of the end-capped acceptor tetra-cyano combined with extended conjugation. Overall, DDHF-DDTC are appropriate donor moieties for their usage in the OSCs applications.

## Materials and methods

Gaussian 09 package^53^ was utilized to perform the calculations. Initially, GaussView 5.0 program^54^was used to yield three dimensional structures of the molecules and input files for Gaussian 09 package. The geometry optimization of BDD-IN molecule was executed by six DFT based functionals: B3LYP^55^, CAM-B3LYP^56^, MPW1PW91^57^, ωB97XD^58^, LC-BLYP^59^ and M06^60^ along with 6-31G(d,p) basis set. Later, structural optimization using frequency analysis at true minima of potential energy surface, TD-DFT calculations were employed for calculating the absorption spectra (λ_max_) of BDD-IN molecule at same levels of theory and basis set combinations. Among all tested functionals, λ_max_ result of M06/6-31G(d,p) functional was found in agreement to the experimental λ_max_ results for BDD-IN molecule. Therefore, M06/6-31G(d,p) level of theory was considered ideal to be used in this study for computing density of state (DOS), TDM surfaces, FMO analysis, reorganization energies, charge transfer analysis, open circuit voltage (V_oc_)_,_ and band gap of BDD-IN as well as designed DDHF, DMDH, DMDC, DDTF and DDTC compounds. The chloroform solvent with conductor-like polarizable continuum (CPCM) model^61^ was utilized for estimating λ_max_values of the investigated compounds.

Reorganization energy was also computed at M06/6-31G (d, p) level of theory. External (λ_ext_) reorganization energy specifies external environmental relaxation, while internal (λ_int_) reorganization energy gives the glance of quick changes in internal composition. In this study only internal environmental effects were focused, and electron (λ_e_), hole (λ_h_) energies were computed employing Eqs. (3) and (4)^62–64^.

3 $$ \lambda_{e} = \left[ {E_{0}^{ - } - E_{ - } } \right] + \left[ {E_{ - }^{0} + E_{0} } \right] $$

4 $$ {\uplambda }_{{\text{h}}} = \left[ {E_{0}^{ + } - E_{ + } } \right] + \left[ {E_{ + }^{0} - E_{0} } \right] $$

where $E_{0}^{ - }$ and $E_{0}^{ + }$ are anionic and cationic energies obtained through optimized structures of neutral molecule, while $E_{ - }^{0}$ and $E_{ + }^{0}$ are molecular energies of neutral molecule calculated at anionic and cationic states. *E*_+_ and *E*_−_ indicate the optimized energies for anionic and cationic structures. *E*_0_ specifies single point ground state energy^46^. The Swizard^65^, PyMOlyze 2.0^66^, Multiwfn 3.7^67^, Origin 8.0, Avogadro^68^ and Chemcraft^69^ programs were used to analyse data.

## Supplementary Information

Supplementary Information.
