# Supplementary material for: Exploration of promising optical and electronic properties of (non-polymer) small donor molecules for organic solar cells
Source: Sci Rep. 2021 Nov 2;11:21540. doi: 10.1038/s41598-021-01070-3 (PMC8564538; doi:10.1038/s41598-021-01070-3)
Supplement: Supplementary file 1 — Supplementary Information. [file 41598_2021_1070_MOESM1_ESM.docx]

## Exploration of Promising Optical and Electronic Properties of Non-Fullerene Based Small Donor Molecules for Organic Solar Cells

Muhammad Khalid,*^1^ Muhammad Usman Khan, ^2^ Saeed Ahmed,^1^ Zahid Shafiq, *^3^ Mohammed Mujahid Alam,^4^ Muhammad Imran,^4^ Ataualpa Albert Carmo Braga^5^, Muhammad Safwan Akram*^6,7^

*^1^Department of Chemistry, Khawaja Fareed University of Engineering & Information Technology, Rahim Yar Khan, 64200, Pakistan*

*^2^Department of Chemistry, University of Okara, Okara-56300, Pakistan*

^3^Institute of Chemical Sciences, Bahauddin Zakariya University, Multan, 60800, Pakistan

*^4^Department of Chemistry, Faculty of Science, King Khalid University, P.O. Box 9004, Abha 61413, Saudi Arabia.*

^5^Departamento de Química Fundamental, Instituto de Química, Universidade de São Paulo, Avenida Professor LineuPrestes, 748, São Paulo 05508-000, Brazil

^6^School of Health and Life Sciences, Teesside University, Middlesbrough, TS1 3BA, UK and ^7^National Horizons Centre, Teesside University, Darlington, DL1 1HG, UK

*Corresponding authors E-mail addresses:

Dr. Muhammad Safwan Akram (Safwan.akram@tees.ac.uk)

Dr. Muhammad Khalid ([muhammad.khalid@kfueit.edu.pk](mailto:muhammad.khalid@kfueit.edu.pk); [Khalid@iq.usp.br](mailto:Khalid@iq.usp.br))

Dr. Muhammad Usman Khan ([usman.chemistry@gmail.com](mailto:usman.chemistry@gmail.com) ; usmankhan@uo.edu.pk)

**Table S1:** Cartesian coordinates of **BDD-IN**

| **Atom** | **X-axis** | **Y-axis** | **Z-axis** |
| --- | --- | --- | --- |
| C | -0.59406400 | 3.46718000 | -0.30791800 |
| C | 0.84289100 | 3.62628600 | -0.17576600 |
| C | 1.38065200 | 4.99794300 | -0.02872200 |
| C | 0.41242600 | 6.10096100 | -0.00026000 |
| C | -1.53553700 | 4.56931300 | -0.30252000 |
| C | -1.01599200 | 2.13841100 | -0.46539800 |
| C | 1.53869800 | 2.41094500 | -0.24544700 |
| C | -0.99151000 | 5.90210700 | -0.12767900 |
| C | -1.73029700 | 7.06789400 | -0.09052800 |
| C | 0.76870900 | 7.42749800 | 0.13564800 |
| S | -0.65521800 | 8.42199500 | 0.04866800 |
| S | 0.37663100 | 1.12312500 | -0.57894200 |
| C | -3.20620600 | 7.29476800 | -0.12207400 |
| H | -3.57332700 | 7.40522900 | -1.15227300 |
| H | -3.71737100 | 6.43605100 | 0.31576400 |
| H | -3.47518700 | 8.20321500 | 0.43078400 |
| C | 2.09478200 | 8.08352800 | 0.36010400 |
| H | 2.75273200 | 7.40293300 | 0.90655300 |
| H | 2.59252500 | 8.32481000 | -0.59054400 |
| H | 1.97813700 | 9.01526800 | 0.92617800 |
| O | -2.75068400 | 4.36460600 | -0.45561200 |
| O | 2.57866000 | 5.24975400 | 0.04152100 |
| C | -2.26988300 | 1.45262700 | -0.53393200 |
| C | -3.71363400 | -0.37597200 | -0.62819200 |
| C | -4.65508600 | 0.65627300 | -0.57864900 |
| H | -3.94840500 | -1.43599700 | -0.69494100 |
| C | 2.85330800 | 1.80232900 | -0.12066900 |
| C | 3.03236100 | 0.45190100 | -0.43572200 |
| H | 2.22710300 | -0.21685700 | -0.73318600 |
| C | 4.32103400 | -0.02682200 | -0.30582200 |
| C | 5.23380100 | 0.92361200 | 0.15061100 |
| H | 4.56283800 | -1.06563800 | -0.51383000 |
| S | 4.40798400 | 2.45070400 | 0.41291300 |
| S | -3.84405900 | 2.23553900 | -0.52625100 |
| C | -2.40738500 | 0.06414900 | -0.59652000 |
| H | -1.56195000 | -0.61765400 | -0.65647400 |
| C | -6.10071200 | 0.48275800 | -0.50190200 |
| C | -7.02528600 | 1.55375100 | -0.57920900 |
| C | -6.65446400 | -0.80220700 | -0.33648800 |
| C | -8.39217600 | 1.32401200 | -0.50217100 |
| C | -8.01987600 | -1.03266000 | -0.25498700 |
| H | -5.98429700 | -1.64711500 | -0.26249300 |
| C | -8.94639800 | 0.03993100 | -0.34229100 |
| H | -9.06198800 | 2.17116400 | -0.56122400 |
| C | 6.66637800 | 0.71364700 | 0.32730900 |
| C | 7.56238900 | 1.66589100 | 0.89696700 |
| C | 7.23788300 | -0.51017700 | -0.08087900 |
| C | 8.91352300 | 1.37545700 | 1.04562100 |
| C | 8.59604800 | -0.79558300 | 0.03586900 |
| H | 6.57798800 | -1.26552900 | -0.49639300 |
| C | 9.48645400 | 0.14887800 | 0.62973400 |
| H | 9.55395700 | 2.12188600 | 1.50012500 |
| O | -6.48470400 | 2.79252300 | -0.71199900 |
| O | 7.00918100 | 2.84878800 | 1.27078800 |
| O | 9.13468300 | -1.95379100 | -0.37455600 |
| O | -8.55099300 | -2.27010000 | -0.10084300 |
| C | 7.59049100 | 3.63684500 | 2.30818800 |
| H | 7.63736900 | 3.07018800 | 3.24962400 |
| H | 8.59285400 | 4.00691500 | 2.04266200 |
| H | 6.91823800 | 4.49381900 | 2.41701100 |
| C | -7.17320400 | 3.81719500 | -1.42040500 |
| H | -7.33398700 | 3.53020500 | -2.46951300 |
| H | -8.13363500 | 4.08257100 | -0.95828200 |
| H | -6.51029800 | 4.68369100 | -1.36934200 |
| C | 10.89724200 | -0.10516100 | 0.81241400 |
| C | 11.83159700 | 0.76808800 | 1.43605000 |
| S | 11.68424200 | -1.57928500 | 0.30782100 |
| C | 13.09330600 | 0.25001900 | 1.55686600 |
| H | 11.58179500 | 1.76914200 | 1.77590000 |
| C | 13.21737200 | -1.05732100 | 1.01386400 |
| H | 13.93081800 | 0.78252500 | 2.00486900 |
| C | -10.39362400 | -0.11529300 | -0.27931700 |
| C | -11.34211500 | 0.88924100 | -0.48607100 |
| S | -11.19818800 | -1.63748000 | 0.04016100 |
| C | -12.65229800 | 0.44774000 | -0.43582200 |
| H | -11.09266600 | 1.92876000 | -0.65570000 |
| C | -12.77705100 | -0.91574300 | -0.16718400 |
| H | -13.51027300 | 1.09682700 | -0.58065800 |
| C | -7.79025700 | -3.33425000 | 0.46642000 |
| H | -8.48736000 | -4.17300300 | 0.52760200 |
| H | -6.94218500 | -3.62324800 | -0.16971400 |
| H | -7.42978300 | -3.06951700 | 1.47030800 |
| C | 8.60879300 | -2.69322800 | -1.61398900 |
| H | 7.91609500 | -3.41910100 | -1.00356900 |
| H | 7.93428700 | -1.94210600 | -2.17658500 |
| H | 9.52231300 | -3.24442800 | -1.88435300 |
| C | 14.30846500 | -1.88050700 | 1.01701500 |
| H | 15.17850700 | -1.45483200 | 1.54563100 |
| C | -14.01766700 | -1.58599700 | -0.14409300 |
| H | -14.85552800 | -0.93541300 | -0.43270000 |
| C | 14.37303700 | -3.19102300 | 0.43020100 |
| C | 13.69185800 | -4.37693300 | 0.96150000 |
| C | 14.98949400 | -3.49770900 | -0.85270000 |
| C | 13.96881600 | -5.47721400 | -0.02930700 |
| C | -14.39632500 | -2.86318800 | 0.14983100 |
| C | -13.62439400 | -4.03257000 | 0.62593600 |
| C | -15.83822000 | -3.26060000 | 0.02531800 |
| C | -14.62726000 | -5.14264000 | 0.78773800 |
| C | 14.69557000 | -4.96348200 | -1.08949100 |
| C | 15.06677200 | -5.75910700 | -2.15951800 |
| C | 13.58879000 | -6.81435500 | 0.01915300 |
| C | 14.68927700 | -7.11762600 | -2.12776900 |
| H | 15.63903900 | -5.33851300 | -2.98835100 |
| C | 13.96069400 | -7.63767900 | -1.05420000 |
| H | 13.01362600 | -7.20300100 | 0.86145100 |
| H | 14.97401100 | -7.77285900 | -2.94900600 |
| H | 13.67708700 | -8.69429600 | -1.04707200 |
| C | -15.90410000 | -4.70039600 | 0.43790900 |
| C | -17.01171400 | -5.55404700 | 0.50361000 |
| C | -14.40725700 | -6.45613800 | 1.22202000 |
| C | -16.80032100 | -6.87239900 | 0.93627200 |
| H | -18.00705700 | -5.19429400 | 0.22560700 |
| C | -15.51246700 | -7.31825800 | 1.29213100 |
| H | -13.40036700 | -6.78971400 | 1.49603500 |
| H | -17.64657100 | -7.56497300 | 1.00031100 |
| H | -15.37361800 | -8.35124900 | 1.62700600 |
| O | 15.58117200 | -2.76554100 | -1.63647200 |
| O | 13.05611100 | -4.52406400 | 1.99432300 |
| O | -12.42444900 | -4.11073400 | 0.85710800 |
| O | -16.77079200 | -2.56253800 | -0.33566800 |

**Table S2:** Cartesian coordinates of **DDHF**.

| **Atom** | | **X-axis** | | | **Y-axis** | **Z-axis** |
| --- | --- | --- | --- | --- | --- | --- |
| C | | 0.70172600 | | 3.03964100 | | 0.44466100 |
| C | | -0.74060900 | | 3.01866600 | | 0.39006800 |
| C | | -1.54059400 | | 4.23714100 | | 0.58921800 |
| C | | -0.79143700 | | 5.43426300 | | 0.98157700 |
| C | | 1.44864100 | | 4.28132500 | | 0.69678200 |
| C | | 1.28550800 | | 1.79079500 | | 0.28274200 |
| C | | -1.27380500 | | 1.75442400 | | 0.18463300 |
| C | | 0.63845400 | | 5.45560100 | | 1.03355300 |
| C | | 1.15635600 | | 6.67196600 | | 1.40347600 |
| C | | -1.37078000 | | 6.63495300 | | 1.30935600 |
| S | | -0.13695300 | | 7.79207200 | | 1.69811500 |
| S | | 0.03222500 | | 0.60697900 | | -0.00811100 |
| C | | 2.57575000 | | 7.08984500 | | 1.55572000 |
| H | | 3.13358100 | | 6.34338500 | | 2.12909100 |
| H | | 3.06624200 | | 7.15268500 | | 0.57736400 |
| H | | 2.65843300 | | 8.06315700 | | 2.04910900 |
| C | | -2.80940000 | | 7.00976200 | | 1.35824800 |
| H | | -3.25954800 | | 6.93745200 | | 0.36228000 |
| H | | -3.36792300 | | 6.31550400 | | 1.99431900 |
| H | | -2.94716100 | | 8.02769800 | | 1.73541800 |
| O | | 2.67313400 | | 4.34626100 | | 0.62990800 |
| O | | -2.75894000 | | 4.26509400 | | 0.43854500 |
| C | | 2.63199000 | | 1.26187700 | | 0.31821700 |
| C | | 4.27668100 | | -0.39294900 | | 0.39512200 |
| C | | 5.07751200 | | 0.71196600 | | 0.16183500 |
| H | | 4.65954700 | | -1.39911900 | | 0.53324000 |
| C | | -2.60419300 | | 1.18825300 | | 0.11801800 |
| C | | -2.85822100 | | -0.17057000 | | 0.23314000 |
| H | | -2.08662200 | | -0.89847200 | | 0.46840600 |
| C | | -4.19621600 | | -0.51750800 | | 0.03058100 |
| C | | -5.00768500 | | 0.56683600 | | -0.25095800 |
| H | | -4.55442600 | | -1.54112300 | | 0.07905600 |
| S | | -4.08256400 | | 2.04405600 | | -0.24887500 |
| S | | 4.11043700 | | 2.16214200 | | 0.08963900 |
| C | | 2.91677500 | | -0.08636700 | | 0.47845100 |
| H | | 2.15251900 | | -0.83222100 | | 0.67908900 |
| C | | 6.52812900 | | 0.69556200 | | 0.01574500 |
| C | | 7.31850100 | | 1.86626800 | | -0.03081600 |
| C | | 7.20463500 | | -0.52792500 | | -0.07789500 |
| C | | 8.69531900 | | 1.78616700 | | -0.15121500 |
| C | | 8.58452900 | | -0.60957500 | | -0.19465800 |
| H | | 6.62464900 | | -1.44380800 | | -0.07169500 |
| C | | 9.37395800 | | 0.55988500 | | -0.23880900 |
| H | | 9.27373000 | | 2.70298800 | | -0.15671600 |
| C | | -6.45233500 | | 0.51454400 | | -0.44020800 |
| C | | -7.20099800 | | 1.57162100 | | -1.00444900 |
| C | | -7.15823700 | | -0.63251600 | | -0.05726200 |
| C | | -8.56673500 | | 1.44330700 | | -1.19264800 |
| C | | -8.52808100 | | -0.75970400 | | -0.23982700 |
| H | | -6.61365700 | | -1.43703500 | | 0.42459900 |
| C | | -9.27141900 | | 0.28286900 | | -0.83333700 |
| H | | -9.11958600 | | 2.27530400 | | -1.61415100 |
| O | | 6.64883300 | | 3.03913700 | | 0.05891800 |
| O | | -6.50483300 | | 2.68626100 | | -1.33076400 |
| O | | -9.22670500 | | -1.85508900 | | 0.13263200 |
| O | | 9.25096200 | | -1.78226200 | | -0.28082000 |
| C | | -7.19240800 | | 3.79019600 | | -1.86727200 |
| H | | -7.67045600 | | 3.54089000 | | -2.82486700 |
| H | | -7.95401200 | | 4.16761400 | | -1.17079800 |
| H | | -6.44267700 | | 4.56542600 | | -2.03215200 |
| C | | 7.36665200 | | 4.24854100 | | 0.03007600 |
| H | | 8.07595500 | | 4.31594700 | | 0.86656000 |
| H | | 7.91059100 | | 4.37143900 | | -0.91675900 |
| H | | 6.62485500 | | 5.04334300 | | 0.12433100 |
| C | | -10.70276500 | | 0.20720900 | | -1.07916900 |
| C | | -11.43021100 | | 1.11096600 | | -1.85184100 |
| S | | -11.73802300 | | -1.02301400 | | -0.41134500 |
| C | | -12.78718400 | | 0.82812700 | | -1.89008000 |
| H | | -10.97953900 | | 1.93897100 | | -2.38800900 |
| C | | -13.15367900 | | -0.30035600 | | -1.15253300 |
| H | | -13.52136500 | | 1.41014800 | | -2.44180400 |
| C | | 10.82210000 | | 0.54278400 | | -0.37179100 |
| C | | 11.62296200 | | 1.66146600 | | -0.59803600 |
| S | | 11.78803000 | | -0.89887100 | | -0.22287200 |
| C | | 12.97702200 | | 1.36498000 | | -0.63640100 |
| H | | 11.23084100 | | 2.66173700 | | -0.74670800 |
| C | | 13.26980800 | | 0.01263200 | | -0.44273100 |
| H | | 13.76158200 | | 2.09868300 | | -0.80498800 |
| C | 8.53415900 | | | -2.99315100 | | -0.22113200 |
| H | 9.27690500 | | | -3.78844400 | | -0.29806200 |
| H | 7.99366000 | | | -3.09218000 | | 0.72995800 |
| H | 7.82295500 | | | -3.07991600 | | -1.05368900 |
| C | -8.54952400 | | | -2.93810100 | | 0.72634800 |
| H | -7.78419500 | | | -3.34782400 | | 0.05322200 |
| H | -8.07975400 | | | -2.64685800 | | 1.67548600 |
| H | -9.30621600 | | | -3.70005100 | | 0.91832600 |
| C | -14.50941200 | | | -0.69951200 | | -1.10710600 |
| H | -15.11962500 | | | -0.01646400 | | -1.69505000 |
| C | 14.61451800 | | | -0.42484800 | | -0.46244100 |
| H | 15.27974300 | | | 0.41859100 | | -0.63639500 |
| C | -15.18606600 | | | -1.72496000 | | -0.49160900 |
| C | -14.58074500 | | | -2.76420300 | | 0.35914500 |
| C | -16.62218700 | | | -1.97415100 | | -0.55129600 |
| C | -15.68660600 | | | -3.62845000 | | 0.81273400 |
| C | 15.22425900 | | | -1.64700000 | | -0.30776700 |
| C | 14.53106100 | | | -2.92175700 | | -0.05550400 |
| C | 16.65742600 | | | -1.91839900 | | -0.35450200 |
| C | 15.57835400 | | | -3.95446400 | | 0.04769800 |
| C | -16.89534300 | | | -3.16665200 | | 0.28015300 |
| C | -18.07803100 | | | -3.84099200 | | 0.58787900 |
| C | -15.59766500 | | -4.73386100 | | | 1.63998400 |
| C | -17.99224900 | | -4.94698800 | | | 1.41604600 |
| H | -19.05752700 | | -3.55240900 | | | 0.22152500 |
| C | -16.77418600 | | -5.39415300 | | | 1.93860300 |
| H | -14.64444600 | | -5.07029300 | | | 2.03831500 |
| C | 16.83661100 | | -3.36971100 | | | -0.13039300 |
| C | 17.97555000 | | -4.17433400 | | | -0.07494200 |
| C | 15.39747300 | | -5.30592800 | | | 0.28082100 |
| C | 17.79837200 | | -5.52788000 | | | 0.15730800 |
| H | 18.98815000 | | -3.80736700 | | | -0.20221700 |
| C | 16.53142300 | | -6.09398400 | | | 0.33387600 |
| H | 14.40706500 | | -5.73155700 | | | 0.41636900 |
| O | -13.40560700 | | -2.90983000 | | | 0.65152800 |
| O | 13.33259400 | | -3.12059800 | | | 0.05297800 |
| C | -17.32149900 | | -0.12225300 | | | -2.03805100 |
| C | -18.96839300 | | -1.62751000 | | | -1.21094200 |
| C | -17.58838800 | | -1.27060700 | | | -1.23593600 |
| N | -17.12609100 | | 0.81769800 | | | -2.69756600 |
| N | -20.10063100 | | -1.89911200 | | | -1.20617000 |
| C | 17.69286200 | | -1.03414100 | | | -0.56193400 |
| C | 17.51534100 | | 0.36411300 | | | -0.77714600 |
| C | 19.06072100 | | -1.43573600 | | | -0.58079900 |
| N | 20.18427800 | | -1.74063900 | | | -0.60041400 |
| N | 17.39100500 | | 1.50852200 | | | -0.95470100 |
| F | 18.85335000 | | -6.32853100 | | | 0.21713500 |
| F | 16.44536200 | | -7.39874200 | | | 0.55147600 |
| F | -16.77738500 | | -6.46197600 | | | 2.72374000 |
| F | -19.09035400 | | -5.61750900 | | | 1.73559000 |

**Table S3:** Cartesian coordinates of **DMDH**.

| **Atom** | **X-axis** | **Y-axis** | **Z-axis** |
| --- | --- | --- | --- |
| C | 0.70172600 | 3.03964100 | 0.44466100 |
| C | -0.74060900 | 3.01866600 | 0.39006800 |
| C | -1.54059400 | 4.23714100 | 0.58921800 |
| C | -0.79143700 | 5.43426300 | 0.98157700 |
| C | 1.44864100 | 4.28132500 | 0.69678200 |
| C | 1.28550800 | 1.79079500 | 0.28274200 |
| C | -1.27380500 | 1.75442400 | 0.18463300 |
| C | 0.63845400 | 5.45560100 | 1.03355300 |
| C | 1.15635600 | 6.67196600 | 1.40347600 |
| C | -1.37078000 | 6.63495300 | 1.30935600 |
| S | -0.13695300 | 7.79207200 | 1.69811500 |
| S | 0.03222500 | 0.60697900 | -0.00811100 |
| C | 2.57575000 | 7.08984500 | 1.55572000 |
| H | 3.13358100 | 6.34338500 | 2.12909100 |
| H | 3.06624200 | 7.15268500 | 0.57736400 |
| H | 2.65843300 | 8.06315700 | 2.04910900 |
| C | -2.80940000 | 7.00976200 | 1.35824800 |
| H | -3.25954800 | 6.93745200 | 0.36228000 |
| H | -3.36792300 | 6.31550400 | 1.99431900 |
| H | -2.94716100 | 8.02769800 | 1.73541800 |
| O | 2.67313400 | 4.34626100 | 0.62990800 |
| O | -2.75894000 | 4.26509400 | 0.43854500 |
| C | 2.63199000 | 1.26187700 | 0.31821700 |
| C | 4.27668100 | -0.39294900 | 0.39512200 |
| C | 5.07751200 | 0.71196600 | 0.16183500 |
| H | 4.65954700 | -1.39911900 | 0.53324000 |
| C | -2.60419300 | 1.18825300 | 0.11801800 |
| C | -2.85822100 | -0.17057000 | 0.23314000 |
| H | -2.08662200 | -0.89847200 | 0.46840600 |
| C | -4.19621600 | -0.51750800 | 0.03058100 |
| C | -5.00768500 | 0.56683600 | -0.25095800 |
| H | -4.55442600 | -1.54112300 | 0.07905600 |
| S | -4.08256400 | 2.04405600 | -0.24887500 |
| S | 4.11043700 | 2.16214200 | 0.08963900 |
| C | 2.91677500 | -0.08636700 | 0.47845100 |
| H | 2.15251900 | -0.83222100 | 0.67908900 |
| C | 6.52812900 | 0.69556200 | 0.01574500 |
| C | 7.31850100 | 1.86626800 | -0.03081600 |
| C | 7.20463500 | -0.52792500 | -0.07789500 |
| C | 8.69531900 | 1.78616700 | -0.15121500 |
| C | 8.58452900 | -0.60957500 | -0.19465800 |
| H | 6.62464900 | -1.44380800 | -0.07169500 |
| C | 9.37395800 | 0.55988500 | -0.23880900 |
| H | 9.27373000 | 2.70298800 | -0.15671600 |
| C | -6.45233500 | 0.51454400 | -0.44020800 |
| C | -7.20099800 | 1.57162100 | -1.00444900 |
| C | -7.15823700 | -0.63251600 | -0.05726200 |
| C | -8.56673500 | 1.44330700 | -1.19264800 |
| C | -8.52808100 | -0.75970400 | -0.23982700 |
| H | -6.61365700 | -1.43703500 | 0.42459900 |
| C | -9.27141900 | 0.28286900 | -0.83333700 |
| H | -9.11958600 | 2.27530400 | -1.61415100 |
| O | 6.64883300 | 3.03913700 | 0.05891800 |
| O | -6.50483300 | 2.68626100 | -1.33076400 |
| O | -9.22670500 | -1.85508900 | 0.13263200 |
| O | 9.25096200 | -1.78226200 | -0.28082000 |
| C | -7.19240800 | 3.79019600 | -1.86727200 |
| H | -7.67045600 | 3.54089000 | -2.82486700 |
| H | -7.95401200 | 4.16761400 | -1.17079800 |
| H | -6.44267700 | 4.56542600 | -2.03215200 |
| C | 7.36665200 | 4.24854100 | 0.03007600 |
| H | 8.07595500 | 4.31594700 | 0.86656000 |
| H | 7.91059100 | 4.37143900 | -0.91675900 |
| H | 6.62485500 | 5.04334300 | 0.12433100 |
| C | -10.70276500 | 0.20720900 | -1.07916900 |
| C | -11.43021100 | 1.11096600 | -1.85184100 |
| S | -11.73802300 | -1.02301400 | -0.41134500 |
| C | -12.78718400 | 0.82812700 | -1.89008000 |
| H | -10.97953900 | 1.93897100 | -2.38800900 |
| C | -13.15367900 | -0.30035600 | -1.15253300 |
| H | -13.52136500 | 1.41014800 | -2.44180400 |
| C | 10.82210000 | 0.54278400 | -0.37179100 |
| C | 11.62296200 | 1.66146600 | -0.59803600 |
| S | 11.78803000 | -0.89887100 | -0.22287200 |
| C | 12.97702200 | 1.36498000 | -0.63640100 |
| H | 11.23084100 | 2.66173700 | -0.74670800 |
| C | 13.26980800 | 0.01263200 | -0.44273100 |
| H | 13.76158200 | 2.09868300 | -0.80498800 |
| C | 8.53415900 | -2.99315100 | -0.22113200 |
| H | 9.27690500 | -3.78844400 | -0.29806200 |
| H | 7.99366000 | -3.09218000 | 0.72995800 |
| H | 7.82295500 | -3.07991600 | -1.05368900 |
| C | -8.54952400 | -2.93810100 | 0.72634800 |
| H | -7.78419500 | -3.34782400 | 0.05322200 |
| H | -8.07975400 | -2.64685800 | 1.67548600 |
| H | -9.30621600 | -3.70005100 | 0.91832600 |
| C | -14.50941200 | -0.69951200 | -1.10710600 |
| H | -15.11962500 | -0.01646400 | -1.69505000 |
| C | 14.61451800 | -0.42484800 | -0.46244100 |
| H | 15.27974300 | 0.41859100 | -0.63639500 |
| C | -15.18606600 | -1.72496000 | -0.49160900 |
| C | -14.58074500 | -2.76420300 | 0.35914500 |
| C | -16.62218700 | -1.97415100 | -0.55129600 |
| C | -15.68660600 | -3.62845000 | 0.81273400 |
| C | 15.22425900 | -1.64700000 | -0.30776700 |
| C | 14.53106100 | -2.92175700 | -0.05550400 |
| C | 16.65742600 | -1.91839900 | -0.35450200 |
| C | 15.57835400 | -3.95446400 | 0.04769800 |
| C | -16.89534300 | -3.16665200 | 0.28015300 |
| C | -18.07803100 | -3.84099200 | 0.58787900 |
| C | -15.59766500 | -4.73386100 | 1.63998400 |
| C | -17.99224900 | -4.94698800 | 1.41604600 |
| H | -19.05752700 | -3.55240900 | 0.22152500 |
| C | -16.77418600 | -5.39415300 | 1.93860300 |
| H | -14.64444600 | -5.07029300 | 2.03831500 |
| C | 16.83661100 | -3.36971100 | -0.13039300 |
| C | 17.97555000 | -4.17433400 | -0.07494200 |
| C | 15.39747300 | -5.30592800 | 0.28082100 |
| C | 17.79837200 | -5.52788000 | 0.15730800 |
| H | 18.98815000 | -3.80736700 | -0.20221700 |
| C | 16.53142300 | -6.09398400 | 0.33387600 |
| H | 14.40706500 | -5.73155700 | 0.41636900 |
| O | -13.40560700 | -2.90983000 | 0.65152800 |
| O | 13.33259400 | -3.12059800 | 0.05297800 |
| C | -17.32149900 | -0.12225300 | -2.03805100 |
| C | -18.96839300 | -1.62751000 | -1.21094200 |
| C | -17.58838800 | -1.27060700 | -1.23593600 |
| N | -17.12609100 | 0.81769800 | -2.69756600 |
| N | -20.10063100 | -1.89911200 | -1.20617000 |
| C | 17.69286200 | -1.03414100 | -0.56193400 |
| C | 17.51534100 | 0.36411300 | -0.77714600 |
| C | 19.06072100 | -1.43573600 | -0.58079900 |
| N | 20.18427800 | -1.74063900 | -0.60041400 |
| N | 17.39100500 | 1.50852200 | -0.95470100 |
| F | 18.85335000 | -6.32853100 | 0.21713500 |
| F | 16.44536200 | -7.39874200 | 0.55147600 |
| F | -16.77738500 | -6.46197600 | 2.72374000 |
| F | -19.09035400 | -5.61750900 | 1.73559000 |

**Table S4:** Cartesian coordinates of **DMDC**.

| **Atom** | **X-axis** | **Y-axis** | **Z-axis** |
| --- | --- | --- | --- |
| C | -0.71869400 | -3.43554100 | 0.31305400 |
| C | 0.72490900 | -3.42975900 | 0.28410500 |
| C | 1.50708000 | -4.65864300 | 0.48824700 |
| C | 0.73851600 | -5.85263300 | 0.85041400 |
| C | -1.48298100 | -4.67062800 | 0.54491300 |
| C | -1.28627300 | -2.17974800 | 0.14656000 |
| C | 1.27606200 | -2.17027700 | 0.09239300 |
| C | -0.69177900 | -5.85842900 | 0.87695500 |
| C | -1.23013000 | -7.07513400 | 1.21522900 |
| C | 1.29861800 | -7.06497100 | 1.16900000 |
| S | 0.04487600 | -8.21408500 | 1.51450700 |
| S | -0.01515000 | -1.00856500 | -0.11863900 |
| C | -2.65635600 | -7.47933900 | 1.33758600 |
| H | -3.18999200 | -6.80855100 | 2.01862100 |
| H | -3.16296000 | -7.39802100 | 0.36969100 |
| H | -2.75293900 | -8.50671100 | 1.70176500 |
| C | 2.73144700 | -7.45822800 | 1.23788900 |
| H | 3.20037900 | -7.37747300 | 0.25122400 |
| H | 3.28663800 | -6.78086200 | 1.89465300 |
| H | 2.84964700 | -8.48337300 | 1.60198000 |
| O | -2.70743600 | -4.72084900 | 0.46548300 |
| O | 2.72815400 | -4.69857300 | 0.36391700 |
| C | -2.62688400 | -1.63529600 | 0.16599000 |
| C | -4.25422800 | 0.03776500 | 0.22849700 |
| C | -5.06745200 | -1.06119400 | 0.00959600 |
| H | -4.62540500 | 1.04965600 | 0.35613600 |
| C | 2.61312500 | -1.61715300 | 0.05563200 |
| C | 2.87558800 | -0.25753700 | 0.14537100 |
| H | 2.10202300 | 0.48467400 | 0.32234400 |
| C | 4.22496000 | 0.07244600 | 0.00285500 |
| C | 5.04014300 | -1.02632900 | -0.20335400 |
| H | 4.58806200 | 1.09458700 | 0.04404500 |
| S | 4.10029100 | -2.49473600 | -0.21327600 |
| S | -4.11527500 | -2.52172600 | -0.05345200 |
| C | -2.89772400 | -0.28231100 | 0.31235100 |
| H | -2.12606900 | 0.45822800 | 0.50419700 |
| C | -6.51902200 | -1.03305500 | -0.12428700 |
| C | -7.31837100 | -2.19802200 | -0.17044300 |
| C | -7.18798800 | 0.19577900 | -0.20245800 |
| C | -8.69565500 | -2.10743400 | -0.27528500 |
| C | -8.56848700 | 0.28805000 | -0.30292400 |
| H | -6.60201200 | 1.10777000 | -0.19438400 |
| C | -9.36650700 | -0.87571900 | -0.34651100 |
| H | -9.28061600 | -3.02009900 | -0.27996800 |
| C | 6.49394400 | -0.99614400 | -0.30688700 |
| C | 7.26366700 | -2.09180100 | -0.75960700 |
| C | 7.19025600 | 0.16596400 | 0.04666900 |
| C | 8.64143000 | -1.99561800 | -0.84757500 |
| C | 8.57130300 | 0.26516400 | -0.04592600 |
| H | 6.62709400 | 1.00920500 | 0.43075600 |
| C | 9.34196200 | -0.83071400 | -0.49235300 |
| H | 9.20149600 | -2.84737000 | -1.21555900 |
| O | -6.65668800 | -3.37625000 | -0.09603800 |
| O | 6.57184100 | -3.20688900 | -1.09289300 |
| O | 9.25654400 | 1.38010300 | 0.28959200 |
| O | -9.22752900 | 1.46580000 | -0.37120300 |
| C | 7.27449000 | -4.35099500 | -1.51316100 |
| H | 7.82378900 | -4.16598400 | -2.44680100 |
| H | 7.97712500 | -4.69824500 | -0.74291000 |
| H | 6.52323000 | -5.12293600 | -1.68661600 |
| C | -7.38422500 | -4.58006300 | -0.11750500 |
| H | -8.08186200 | -4.64366500 | 0.72901200 |
| H | -7.94278100 | -4.69606900 | -1.05663800 |
| H | -6.64722900 | -5.38067600 | -0.03584300 |
| C | 10.79331700 | -0.80700800 | -0.57827100 |
| C | 11.59844300 | -1.90387100 | -0.88680300 |
| S | 11.75641300 | 0.61991300 | -0.31162500 |
| C | 12.95003800 | -1.59983600 | -0.91278400 |
| H | 11.21468200 | -2.90107300 | -1.07159400 |
| C | 13.23934400 | -0.26304200 | -0.62478200 |
| H | 13.73734000 | -2.31917900 | -1.12489600 |
| C | -10.81554900 | -0.84783000 | -0.46147400 |
| C | -11.62641100 | -1.95943400 | -0.69033200 |
| S | -11.76976200 | 0.59801900 | -0.28369700 |
| C | -12.97839600 | -1.65441900 | -0.70745300 |
| H | -11.24186500 | -2.95996200 | -0.85585100 |
| C | -13.26059600 | -0.30208800 | -0.49390600 |
| H | -13.76965700 | -2.38127800 | -0.87434300 |
| C | -8.50051500 | 2.67089800 | -0.31142400 |
| H | -9.23806600 | 3.47256800 | -0.36862200 |
| H | -7.94381500 | 2.75589300 | 0.63159300 |
| H | -7.80207400 | 2.75954600 | -1.15447900 |
| C | 8.55724900 | 2.51869700 | 0.73585300 |
| H | 7.84710000 | 2.87539400 | -0.02240300 |
| H | 8.01874100 | 2.31736900 | 1.67176500 |
| H | 9.31160100 | 3.28675200 | 0.91229300 |
| C | 14.58318200 | 0.17238000 | -0.60088700 |
| H | 15.24793400 | -0.65540800 | -0.84002000 |
| C | -14.60199100 | 0.14225100 | -0.48903000 |
| H | -15.27424300 | -0.69538900 | -0.66365900 |
| C | 15.19502000 | 1.37779400 | -0.34564200 |
| C | 14.50056200 | 2.63260700 | -0.01428900 |
| C | 16.62904900 | 1.64410300 | -0.35480800 |
| C | 15.55059800 | 3.65371300 | 0.16863600 |
| C | -15.20352100 | 1.36650900 | -0.30947400 |
| C | -14.49772500 | 2.63187100 | -0.04952600 |
| C | -16.63548100 | 1.64515300 | -0.33055100 |
| C | -15.53825100 | 3.66921700 | 0.08639900 |
| C | 16.80942200 | 3.07722100 | -0.03031700 |
| C | 17.94392200 | 3.87291600 | 0.09617100 |
| C | 15.37794500 | 4.98405700 | 0.48607200 |
| C | 17.78380600 | 5.22107300 | 0.41695700 |
| H | 18.95086800 | 3.49430000 | -0.04345500 |
| C | 16.51151400 | 5.78053100 | 0.61192000 |
| H | 14.38235500 | 5.39489500 | 0.63241600 |
| C | -16.80259000 | 3.09503700 | -0.08092300 |
| C | -17.92883800 | 3.90782000 | 0.00490800 |
| C | -15.35189500 | 5.01194900 | 0.33686600 |
| C | -17.75489000 | 5.26891300 | 0.25661800 |
| H | -18.93970900 | 3.53338300 | -0.11517000 |
| C | -16.47715200 | 5.82489400 | 0.42357200 |
| H | -14.35208500 | 5.41935900 | 0.46222100 |
| O | 13.30136500 | 2.82648200 | 0.09414800 |
| O | -13.29661000 | 2.82224100 | 0.04248200 |
| C | 17.48259500 | -0.60972500 | -0.91480100 |
| C | 19.03412900 | 1.16302200 | -0.57810100 |
| C | 17.66387700 | 0.77046200 | -0.60569900 |
| N | 17.35474100 | -1.73926800 | -1.16876000 |
| N | 20.16059400 | 1.45713500 | -0.56119900 |
| C | -17.67865400 | 0.76981300 | -0.53662700 |
| C | -17.51054700 | -0.62541200 | -0.77795100 |
| C | -19.04511800 | 1.17644100 | -0.52740400 |
| N | -20.16851800 | 1.48244200 | -0.52435400 |
| N | -17.39306100 | -1.76710800 | -0.97639600 |
| Cl | -19.16580100 | 6.26590600 | 0.35686200 |
| Cl | -16.27127100 | 7.51418600 | 0.73677000 |
| Cl | 16.32214700 | 7.45377500 | 1.00940300 |
| Cl | 19.20455700 | 6.19695200 | 0.56837900 |

**Table S5:** Cartesian coordinates of **DDTF**.

| **Atom** | **X-axis** | **Y-axis** | **Z-axis** |
| --- | --- | --- | --- |
| C | -0.71127600 | -3.27240300 | 0.45701900 |
| C | 0.73079300 | -3.25791200 | 0.40646300 |
| C | 1.52485400 | -4.47965400 | 0.61326100 |
| C | 0.76968000 | -5.67234200 | 1.00546300 |
| C | -1.46444600 | -4.50974000 | 0.71591800 |
| C | -1.28889100 | -2.02195100 | 0.28610300 |
| C | 1.27002800 | -1.99693300 | 0.19476300 |
| C | -0.66025500 | -5.68675700 | 1.05379600 |
| C | -1.18529500 | -6.90213600 | 1.41719100 |
| C | 1.34220900 | -6.87671200 | 1.33250000 |
| S | 0.10145300 | -8.02866000 | 1.71309200 |
| S | -0.03027500 | -0.84508300 | -0.00759000 |
| C | -2.60687000 | -7.31242700 | 1.56842000 |
| H | -3.13008600 | -6.64125200 | 2.25723900 |
| H | -3.13203600 | -7.23643100 | 0.61012700 |
| H | -2.69201000 | -8.33854400 | 1.93870900 |
| C | 2.77855600 | -7.25934900 | 1.38479900 |
| H | 3.23299900 | -7.18351400 | 0.39106200 |
| H | 3.33808300 | -6.57249500 | 2.02797900 |
| H | 2.90936500 | -8.28025800 | 1.75622400 |
| O | -2.68941600 | -4.56778900 | 0.65323000 |
| O | 2.74350500 | -4.51239600 | 0.46667400 |
| C | -2.63367200 | -1.48758300 | 0.31404600 |
| C | -4.27134400 | 0.17438900 | 0.37302400 |
| C | -5.07607900 | -0.92991100 | 0.14856400 |
| H | -4.64969000 | 1.18355800 | 0.50092700 |
| C | 2.60350100 | -1.43719300 | 0.12863900 |
| C | 2.86254600 | -0.07749900 | 0.22647900 |
| H | 2.09229000 | 0.65782600 | 0.44199100 |
| C | 4.20412000 | 0.26012000 | 0.03566300 |
| C | 5.01406600 | -0.83244000 | -0.21915600 |
| H | 4.56531200 | 1.28305500 | 0.07451500 |
| S | 4.08107100 | -2.30519600 | -0.20762900 |
| S | -4.11406200 | -2.38450800 | 0.09099900 |
| C | -2.91320900 | -0.13648700 | 0.46160000 |
| H | -2.14660300 | 0.60843700 | 0.65636500 |
| C | -6.52595100 | -0.90908700 | 0.00264700 |
| C | -7.31996800 | -2.07898400 | -0.04553500 |
| C | -7.19864100 | 0.31624200 | -0.09025900 |
| C | -8.69557000 | -1.99546000 | -0.16700300 |
| C | -8.57825500 | 0.40196000 | -0.20698700 |
| H | -6.61677600 | 1.23076900 | -0.08202000 |
| C | -9.37134600 | -0.76601300 | -0.25206800 |
| H | -9.27511900 | -2.91142100 | -0.17621100 |
| C | 6.46083700 | -0.79141600 | -0.38826900 |
| C | 7.21321600 | -1.87026000 | -0.90879000 |
| C | 7.16724500 | 0.36295900 | -0.03067200 |
| C | 8.58212700 | -1.75814100 | -1.07281000 |
| C | 8.54119100 | 0.47547100 | -0.19133900 |
| H | 6.62023600 | 1.18674000 | 0.41418500 |
| C | 9.29112700 | -0.59309100 | -0.73113200 |
| H | 9.13109400 | -2.60379300 | -1.47033600 |
| O | -6.65248200 | -3.25222200 | 0.04317900 |
| O | 6.51352700 | -2.98567400 | -1.22123100 |
| O | 9.23776000 | 1.57837000 | 0.15586100 |
| O | -9.24060200 | 1.57544500 | -0.29158600 |
| C | 7.19977300 | -4.11137800 | -1.71345200 |
| H | 7.69841800 | -3.89327300 | -2.66809100 |
| H | 7.94324300 | -4.47709900 | -0.99166800 |
| H | 6.44495900 | -4.88293300 | -1.87173900 |
| C | -7.37179600 | -4.46134800 | 0.01701900 |
| H | -8.08079500 | -4.52567400 | 0.85390900 |
| H | -7.91534400 | -4.58560000 | -0.92977100 |
| H | -6.63064900 | -5.25640300 | 0.11324100 |
| C | 10.72758600 | -0.53792100 | -0.93533300 |
| C | 11.49016400 | -1.53092700 | -1.55666600 |
| S | 11.73003800 | 0.78777300 | -0.41661600 |
| C | 12.84003800 | -1.23352500 | -1.60420900 |
| H | 11.07181300 | -2.44080400 | -1.97198300 |
| C | 13.17452100 | -0.00312000 | -1.02481100 |
| H | 13.59548500 | -1.87704000 | -2.04847400 |
| C | -10.81727100 | -0.74268800 | -0.38088600 |
| C | -11.62761300 | -1.86322400 | -0.58413400 |
| S | -11.77184600 | 0.70747800 | -0.25994900 |
| C | -12.97659400 | -1.55975600 | -0.62892100 |
| H | -11.24196500 | -2.86879800 | -0.71080600 |
| C | -13.26222200 | -0.19862600 | -0.46225500 |
| H | -13.76570600 | -2.29183000 | -0.78245800 |
| C | -8.52113800 | 2.78653100 | -0.23618700 |
| H | -9.26300200 | 3.58232600 | -0.31458400 |
| H | -7.98021300 | 2.88665300 | 0.71419200 |
| H | -7.81166200 | 2.86878700 | -1.07027900 |
| C | 8.55839300 | 2.69120900 | 0.69234000 |
| H | 7.81253500 | 3.08078900 | -0.01335200 |
| H | 8.06635500 | 2.44091700 | 1.64154200 |
| H | 9.31897300 | 3.45250600 | 0.87045300 |
| C | 14.51762600 | 0.41907600 | -1.00965500 |
| H | 15.15171700 | -0.32882400 | -1.48178200 |
| C | -14.59870200 | 0.24496300 | -0.48842800 |
| H | -15.27182700 | -0.59516400 | -0.64683800 |
| C | 15.16773900 | 1.54029000 | -0.53568900 |
| C | 14.52457700 | 2.67434200 | 0.13885900 |
| C | 16.59666500 | 1.80657900 | -0.61141000 |
| C | 15.60475600 | 3.63158600 | 0.46717500 |
| C | -15.20119100 | 1.47956500 | -0.35179100 |
| C | -14.49436100 | 2.74376500 | -0.11627000 |
| C | -16.62932600 | 1.76056700 | -0.39996700 |
| C | -15.53320600 | 3.79229300 | -0.01734400 |
| C | 16.83201700 | 3.12614800 | 0.02501900 |
| C | 17.98727900 | 3.87494600 | 0.23083200 |
| C | 15.47593700 | 4.84681100 | 1.10510700 |
| C | 17.87862600 | 5.11194800 | 0.87617600 |
| H | 18.96974800 | 3.54297100 | -0.08817400 |
| C | 16.62962000 | 5.60380300 | 1.31615400 |
| H | 14.50047400 | 5.19792000 | 1.43183000 |
| C | -16.79808000 | 3.21975100 | -0.18588200 |
| C | -17.92319300 | 4.03781400 | -0.13071500 |
| C | -15.33652100 | 5.13811500 | 0.20529100 |
| C | -17.74639200 | 5.40791900 | 0.09413800 |
| H | -18.93281200 | 3.66099000 | -0.25378900 |
| C | -16.45931800 | 5.96500600 | 0.26341300 |
| H | -14.33287700 | 5.53590900 | 0.33146500 |
| O | 13.34450600 | 2.83902100 | 0.39774700 |
| O | -13.29561000 | 2.94171400 | -0.01310500 |
| C | 17.36415500 | -0.24063800 | -1.76653800 |
| C | 18.96608600 | 1.41117400 | -1.15822300 |
| C | 17.59343300 | 1.02669400 | -1.15435600 |
| N | 17.19496800 | -1.27697600 | -2.27018900 |
| N | 20.09310400 | 1.70282600 | -1.17329100 |
| C | -17.67565000 | 0.88824700 | -0.60131900 |
| C | -17.50958400 | -0.51272300 | -0.80867500 |
| C | -19.03970100 | 1.30217200 | -0.62279100 |
| N | -20.16031700 | 1.61703800 | -0.64447700 |
| N | -17.39051500 | -1.65855500 | -0.97925500 |
| C | -16.29595100 | 7.36655800 | 0.49377500 |
| N | -16.13390400 | 8.50168500 | 0.68236200 |
| C | -18.89853000 | 6.25440900 | 0.15311200 |
| N | -19.83726600 | 6.93735100 | 0.20057400 |
| C | 16.53628400 | 6.86895900 | 1.97550100 |
| N | 16.43250500 | 7.89322300 | 2.51439700 |
| C | 19.06211700 | 5.88725100 | 1.09052700 |
| N | 20.02685900 | 6.51185600 | 1.26103800 |

**Table S6:** Cartesian coordinates of **DDTC**.

| **Atom** | **X-axis** | | | **Y-axis** | **Z-axis** |
| --- | --- | --- | --- | --- | --- |
| C | -0.67692700 | | 3.06518100 | | -0.26812100 |
| C | 0.76421800 | | 3.04388100 | | -0.18464800 |
| C | 1.56785300 | | 4.26197800 | | -0.37192000 |
| C | 0.82824400 | | 5.45915300 | | -0.78040200 |
| C | -1.41646900 | | 4.30720100 | | -0.54035500 |
| C | -1.26525400 | | 1.81732500 | | -0.10926700 |
| C | 1.29287500 | | 1.78035000 | | 0.03914200 |
| C | -0.59976400 | | 5.48098800 | | -0.86114300 |
| C | -1.10994900 | | 6.69737500 | | -1.24184600 |
| C | 1.41486300 | | 6.65930600 | | -1.09713700 |
| S | 0.18965800 | | 7.81686200 | | -1.51097900 |
| S | -0.01793600 | | 0.63486000 | | 0.21324700 |
| C | -2.52580800 | | 7.11662300 | | -1.42091400 |
| H | -3.07500100 | | 6.36796600 | | -1.99969900 |
| H | -3.03233800 | | 7.18506500 | | -0.45107200 |
| H | -2.59844100 | | 8.08779800 | | -1.92004900 |
| C | 2.85435600 | | 7.03351900 | | -1.11663600 |
| H | 3.28212600 | | 6.96803000 | | -0.11032200 |
| H | 3.42652400 | | 6.33435900 | | -1.73495000 |
| H | 3.00086100 | | 8.04889800 | | -1.49738900 |
| O | -2.64211200 | | 4.37429900 | | -0.50180900 |
| O | 2.78315100 | | 4.29086900 | | -0.19822900 |
| C | -2.61151400 | | 1.29005900 | | -0.17147800 |
| C | -4.26170900 | | -0.35932900 | | -0.27016500 |
| C | -5.06608500 | | 0.75660400 | | -0.11044700 |
| H | -4.64332900 | | -1.36808100 | | -0.39288500 |
| C | 2.62188300 | | 1.21423100 | | 0.13043900 |
| C | 2.87714800 | | -0.14692900 | | 0.04497500 |
| H | 2.10624200 | | -0.88103100 | | -0.17264700 |
| C | 4.21631700 | | -0.48840500 | | 0.24894000 |
| C | 5.02815900 | | 0.60286900 | | 0.50163400 |
| H | 4.57531600 | | -1.51251000 | | 0.21898100 |
| S | 4.10087000 | | 2.07821700 | | 0.47570400 |
| S | -4.09320100 | | 2.20261000 | | -0.02987800 |
| C | -2.89786000 | | -0.06167200 | | -0.30178000 |
| H | -2.13098900 | -0.81792500 | | | -0.44592400 |
| C | -6.52294300 | 0.75745600 | | | -0.05956300 |
| C | -7.29811900 | 1.94007500 | | | -0.05444100 |
| C | -7.22394700 | -0.45554000 | | | -0.02145900 |
| C | -8.67986200 | 1.88094000 | | | -0.04457700 |
| C | -8.61029400 | -0.51573300 | | | -0.00610700 |
| H | -6.65944000 | -1.38083300 | | | 0.00360000 |
| C | -9.38366700 | 0.66549400 | | | -0.03152600 |
| H | -9.23973900 | 2.80862800 | | | -0.03913000 |
| C | 6.47566800 | 0.56198200 | | | 0.67117000 |
| C | 7.22684100 | 1.63944200 | | | 1.19303600 |
| C | 7.18333600 | -0.58847500 | | | 0.30211600 |
| C | 8.59877600 | 1.53262100 | | | 1.34306800 |
| C | 8.55930100 | -0.69541100 | | | 0.44902800 |
| H | 6.63541400 | -1.41110600 | | | -0.14412700 |
| C | 9.30782400 | 0.37357800 | | | 0.98739000 |
| H | 9.15252000 | 2.38175800 | | | 1.72728900 |
| O | -6.60831900 | 3.10431700 | | | -0.06162300 |
| O | 6.52636400 | 2.75242900 | | | 1.51549100 |
| O | 9.26020300 | -1.79250900 | | | 0.08863100 |
| O | -9.29997900 | -1.67662600 | | | 0.03258300 |
| C | 7.21477200 | 3.87843800 | | | 2.00267900 |
| H | 7.72356100 | 3.66009200 | | | 2.95199800 |
| H | 7.95085100 | 4.24724300 | | | 1.27481700 |
| H | 6.45996900 | 4.64832400 | | | 2.16953700 |
| C | -7.30993100 | 4.32295600 | | | -0.10268900 |
| H | -7.94468700 | 4.38983200 | | | -0.99715100 |
| H | -7.93176900 | 4.45982600 | | | 0.79282100 |
| H | -6.55301600 | 5.10813200 | | | -0.13883600 |
| C | 10.74924600 | 0.32774700 | | | 1.17186200 |
| C | 11.49800600 | 1.27299400 | | | 1.87266200 |
| S | 11.77154900 | -0.91118100 | | | 0.50091100 |
| C | 12.85925500 | 1.01386900 | | | 1.85316600 |
| H | 11.06073000 | 2.11593000 | | | 2.39648100 |
| C | 13.20964700 | -0.13678500 | | | 1.14051000 |
| H | 13.60974100 | 1.63017700 | | | 2.34225700 |
| C | -10.83682100 | 0.67355600 | | | -0.05068800 |
| C | -11.62865800 | 1.81761100 | | | -0.16014300 |
| S | -11.82143400 | -0.76020200 | | | 0.05894800 |
| C | -12.98625000 | 1.54544900 | | | -0.15421600 |
| H | -11.22961500 | 2.82146100 | | | -0.25479400 |
| C | -13.29457400 | 0.18678800 | | | -0.04012500 |
| H | -13.76480600 | 2.30002500 | | | -0.23722200 |
| C | -8.59996200 | -2.89889900 | | | 0.07638600 |
| H | -9.35851200 | -3.68105000 | | | 0.12778100 |
| H | -7.98977600 | -3.04276700 | | | -0.82539100 |
| H | -7.95626100 | -2.95820900 | | | 0.96425300 |
| C | 8.58281900 | -2.89776100 | | | -0.46359700 |
| H | 7.84064600 | -3.30182100 | | | 0.23824700 |
| H | 8.08515600 | -2.63375600 | | | -1.40636100 |
| H | 9.34488400 | -3.65357700 | | | -0.65822400 |
| C | 14.56893400 | -0.50671800 | | | 1.03387900 |
| H | 15.19286200 | 0.21195700 | | | 1.56153900 |
| C | -14.64655900 | -0.21986200 | | | -0.03599100 |
| H | -15.29506500 | 0.64922200 | | | -0.12286600 |
| C | 15.23971000 | -1.53797800 | | | 0.41836400 |
| C | 14.60706000 | -2.62764400 | | | -0.34713900 |
| C | 16.67998800 | -1.74489200 | | | 0.40093500 |
| C | 15.70958500 | -3.48545500 | | | -0.82354000 |
| C | -15.29067000 | -1.43183500 | | | 0.05225600 |
| C | -14.62745800 | -2.73985600 | | | 0.19631300 |
| C | -16.72870500 | -1.64657800 | | | 0.03039300 |
| C | -15.71116200 | -3.74264600 | | | 0.26711700 |
| C | 16.93994400 | -2.97269000 | | | -0.38588300 |
| C | 18.12674900 | -3.62051100 | | | -0.71749200 |
| C | 15.64202500 | -4.63548300 | | | -1.58537000 |
| C | 18.03919900 | -4.77263600 | | | -1.48520400 |
| H | 19.11141100 | -3.28264800 | | | -0.41436500 |
| C | 16.82193200 | -5.29006900 | | | -1.92387900 |
| C | -16.95734300 | -3.10254600 | | | 0.17161300 |
| C | -18.13536500 | -3.84211900 | | | 0.21770200 |
| C | -15.62568700 | -5.11520000 | | | 0.40772100 |
| C | -18.02814200 | -5.21727400 | | | 0.35909600 |
| H | -19.12859600 | -3.41255600 | | | 0.15009300 |
| C | -16.79782300 | -5.86396000 | | | 0.45345400 |
| O | 13.41997700 | -2.80303500 | | | -0.55416500 |
| O | -13.43150300 | -2.96200700 | | | 0.25140100 |
| C | 17.41344300 | 0.20568100 | | | 1.73575100 |
| C | 19.05389400 | -1.29210600 | | | 0.88942400 |
| C | 17.66647200 | -0.97857400 | | | 0.98289200 |
| N | 17.22970900 | 1.17610200 | | | 2.35323000 |
| N | 20.19314300 | -1.52514300 | | | 0.82806600 |
| C | -17.73593500 | -0.71447100 | | | -0.09999200 |
| C | -17.50967700 | 0.68627800 | | | -0.24197600 |
| C | -19.11996700 | -1.05465500 | | | -0.11029000 |
| N | -20.25769400 | -1.30255400 | | | -0.12243200 |
| N | -17.34811500 | 1.83388000 | | | -0.35927500 |
| F | -16.74759700 | -7.17812000 | | | 0.58608000 |
| F | -19.12058700 | -5.96358300 | | | 0.40838000 |
| F | -14.46842400 | -5.74213300 | | | 0.50132900 |
| F | 14.49231800 | -5.13014000 | | | -2.00131700 |
| F | 16.79279500 | -6.39195400 | | | -2.65317600 |
| F | 19.14061200 | -5.42260700 | | | -1.82759800 |
